# Electro‐Synthesis of Organic Compounds with Heterogeneous Catalysis

**DOI:** 10.1002/advs.202205077

**Published:** 2022-11-18

**Authors:** Tariq Ali, Haiyan Wang, Waseem Iqbal, Tariq Bashir, Rahim Shah, Yong Hu

**Affiliations:** ^1^ Key Laboratory of the Ministry of Education for Advanced Catalysis Materials Department of Chemistry Zhejiang Normal University Jinhua 321004 China; ^2^ Dipartimento di Chimica e Tecnologie Chimiche Università della Calabria Rende CS 87036 Italy; ^3^ Jiangsu Provincial Key Laboratory for Advanced Carbon Materials and Wearable Energy Technologies Soochow University Suzhou 215006 China; ^4^ Institute of Chemical Sciences University of Swat Swat Khyber Pakhtunkhwa 19130 Pakistan; ^5^ Hangzhou Institute of Advanced Studies Zhejiang Normal University Hangzhou 311231 China

**Keywords:** electro‐organic synthesis, heterogeneous catalysts, paired electrolysis, reactor engineering, value‐added chemicals

## Abstract

Electro‐organic synthesis has attracted a lot of attention in pharmaceutical science, medicinal chemistry, and future industrial applications in energy storage and conversion. To date, there has not been a detailed review on electro‐organic synthesis with the strategy of heterogeneous catalysis. In this review, the most recent advances in synthesizing value‐added chemicals by heterogeneous catalysis are summarized. An overview of electrocatalytic oxidation and reduction processes as well as paired electrocatalysis is provided, and the anodic oxidation of alcohols (monohydric and polyhydric), aldehydes, and amines are discussed. This review also provides in‐depth insight into the cathodic reduction of carboxylates, carbon dioxide, C=C, C≡C, and reductive coupling reactions. Moreover, the electrocatalytic paired electro‐synthesis methods, including parallel paired, sequential divergent paired, and convergent paired electrolysis, are summarized. Additionally, the strategies developed to achieve high electrosynthesis efficiency and the associated challenges are also addressed. It is believed that electro‐organic synthesis is a promising direction of organic electrochemistry, offering numerous opportunities to develop new organic reaction methods.

## Graphical abstract

In this review, the most recent advances in synthesizing value‐added chemicals by heterogeneous catalysis are summarized, and an overview of electrocatalytic oxidation and reduction processes as well as paired electrocatalysis is also provided.

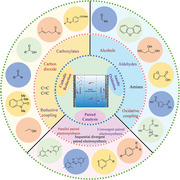

## 1 Introduction

The development and use of new clean energy are receiving significant attention in the modern era of scarce resources, excessive use of fossil energy, and severe environmental pollution.^[^ ^1^ ^]^ As an eco‐friendly and energy‐renewable technique, electro‐organic synthesis is becoming more important for the chemical industry and biomedicine.^[^ ^2^ ^]^ To produce value‐added compounds, electrochemical cells can manipulate redox potential and initiate desired reaction sequences,^[^ ^3^ ^]^ including synthesis of C—C, C—O, C—S, and C—N bonds.^[^ ^4^ ^]^ Differing from other types of organic synthesis, electro‐organic synthesis utilizes electric energy to drive a reaction, which eliminates the need for expensive and toxic chemical oxidants. Another benefit of electro‐organic synthesis is that it can be controlled to a high degree of accuracy. Selectivity of organic reactions can be effectively achieved by controlling the oxidation current and voltage and creating a new method for synthesis reactions that traditional organic synthesis methods cannot reach. Therefore, the development of effective electrocatalysts and the wise selection of cathodic and anodic half‐reactions are important to be considered.

Electrochemical reactions are important for synthesizing complex molecules because they allow the formation of C—C and C–heteroatom bonds without external oxidants. Electrochemical reactions can be classified as electrochemical oxidation reactions at the anode, electrochemical reduction reactions at the cathode, and paired electrocatalytic processes. Traditional oxidative reactions require stoichiometric amounts of oxidizing agents, such as high‐valent chromium and manganese compounds or hypervalent iodine species. Still, electrochemical oxidation reactions allow for milder reactions involving O_2_ and H_2_O_2_.^[^ ^5^ ^]^ The ACS Green Chemistry Institute Pharmaceutical Roundtable identified “alternatives for oxidations” as a critical green chemistry research area in their 2018 report.^[^ ^6^ ^]^ From a green chemistry perspective, using a catalyst regenerated during anodic oxidation is highly desirable since the waste generated is significantly reduced. Specially, oxidative cross‐coupling reactions can be achieved through electrochemical synthesis.^[^ ^7^ ^]^ The electrochemical oxidative cross‐coupling with hydrogen evolution reaction (HER) of anodic substrate oxidation with cathodic proton reduction is one of the most effective methods studied extensively.

In recent decades, the resurgence of total organic electrosynthesis has also sparked increased interest in cathodic reduction‐enabled processes, resulting in various innovative technologies. The use of cathodic reduction for milder conditions and safer sacrificial reductants appears to be more promising than traditional methods that use potentially hazardous materials such as hydrogen, low‐valent metals, metal hydrides, silanes, and boranes. Despite the apparent conceptual appeal, actual procedures that promote high efficiency and selectivity are limited at the preparative scale. This is due to the inherent problems caused by cathodic processes, such as proton/oxygen reduction, which compete with desirable electrode transformations and passivation in severely reducing conditions. This review focuses on the latest developments in cathodic reduction‐enabled organic transformations, emphasizing their fundamental scopes, constraints, applications, and mechanisms of action.

Presently, most electrochemical systems are designed to focus only on one side. The counter‐electrode reaction is engineered with fast dynamics or separated from the primary response by splitting the cell. Therefore, developing technologies that use electric currents on both sides of the cell can enhance energy efficiency. Because the electrochemical industries use the most energy on the earth, paired electrolysis with both half‐cell processes is perfect for creating value‐added compounds. Anodic and cathodic electrocatalysis are applied to all electrochemical reactions that proceed in pairs. Further by separating the reactions in electrolysis over two electrodes, the possibility of establishing a redox‐neutral reaction is presented that would otherwise be impossible within a reaction flask context. Combined electrolysis improves the atom and energy efficiency of electrocatalytic synthesis significantly. Heterogeneous‐catalyzed paired electrolysis is expected to remain an important field of organic electrochemistry for years to come, advancing the development of new organic reaction techniques.

Many review articles have been published on anodic oxidation and cathodic reduction electrochemistry in electro‐organic synthesis.^[^ ^2^, ^3^, ^8^ ^]^ However, there has not been a detailed review of electro‐organic synthesis through heterogeneous catalysis since a comprehensive analysis of modern electro‐synthetic methodologies from 2000 through 2017 has already been completed.^[^ ^3a^ ^]^ Considering the above, this review covers recent developments in electro‐organic synthesis based on heterogeneous catalysts, especially in the latest five years. According to the productive heterogonous electrocatalytic electrodes, this review is divided into three parts: 1) anodic oxidation, 2) cathodic reduction, and 3) paired electrolysis. The anodic oxidations of alcohols, aldehydes, amines, and oxidative coupling reactions are presented systematically. The cathodic reductions of carboxylates, carbon dioxide, C=C, C≡C, and reductive coupling reactions, as well as electrocatalytic paired electro‐synthesis are summarized. Furthermore, we discuss strategies for improving electrosynthesis efficiency while addressing its associated challenges. Eventually, this field is likely to reach widespread adoption among synthetic researchers.

## 2 Advanced Electrocatalysis for Anodic Reactions

**Scheme 1:**
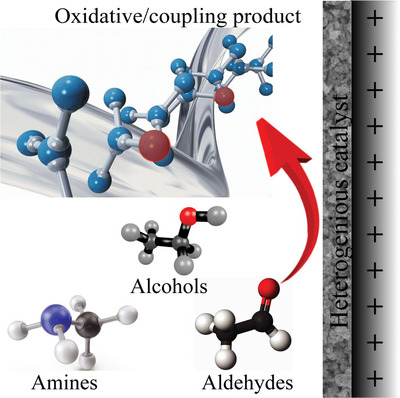
Schematic illustration of electro‐catalytic oxidation over heterogeneous catalysts.

Anodic oxidation generates considerably less waste, which makes the regeneration of catalysts an advantageous approach from an environmental perspective (**Scheme** **1**), and H_2_ gas is a major by‐product of electrochemical oxidations since these reactions usually involve HERs. It can utilize more valuable and practical oxidants such as O_2_ and H_2_O_2_. Anodic oxidation has been regarded as an efficient way to form value‐added chemicals by heteroatoms catalyst (**Table** **1**). Therefore, the following subsections are used to categorize electro‐oxidative reactions.

**Table 1:** Electrocatalytic oxidative performances of various catalysts

Catalyst	Electrolyte	Organic substrate	Organic product	*η*_org._ [V]	FE [%, org.]	Yield [%, org.]	Ref.
Pd/TNTA‐web^a)^	2.0 m NaOH	2 m EtOH	Acetic acid	0.69	—		[9]
F‐*β*‐FeOOH^b)^	1.0 m KOH	EtOH: H_2_O = 15 : 5	Acetic acid	1.207, 1.43	≈72	—	[10]
Au NP‐modified column electrode	H_2_O/acetonitrile solution (80/20 in vol%)	50 mm BA	Benzaldehyde	0.4	78–89	—	[11]
Co^II^P_3_ ^c)^	Bu_4_NBF_4_ or Et_4_NBF_4_ in CH_3_CN	536 mm BA	Benzaldehyde	0.63 *V*_app_	97	99	[12]
NC@CuCo_2_N_x_/CF^d)^	1.0 m KOH	15 mm BA	Benzaldehyde	1.25, 1.55	≈81	—	[13]
MOF‐TEMPO^e)^	CH_3_CN	0.5 mm BA	Benzaldehyde/Benzoic acid	—	—	96	[14]
hp‐Ni^f)^	1 m KOH	10 mm BA	Benzoic acid	1.35, 1.50	97		[15]
NiS_2_/CFC^g)^	1.0 m KOH	0.45 m 2‐propanol	Acetone	1.348	98	—	[16]
[Ru(acac)_2_(pyimN)] (Ru^III^N3)^h)^	0.1 m Bu_4_NBF_4_ in THF with 2‐propanol (0.30 m)	2‐propanol	Acetone	−0.70 V vs Fc^+/0^)	85	—	[17]
Co_3_O_4_ NSs/CP^i)^	1.0 m KOH	1 m EtOH	Ethyl acetate	1.445	98	—	[18]
3D PdCu alloy	1.0 m KOH	1 m EtOH	Ethyl acetate	1.56 *V*_onset_	—	—	[19]
CuO‐NRs^j)^	1 m KOH	Furfuryl alcohol	Furaldehyde/2‐furoic acid	1.35–1.39	98	≥98	[20]
S‐MnO_2_/NF^k)^	1.0 m KOH	0.5 m Urea	Urea conversion	1.33, 1.41	—	—	[21]
Ni_2_P/NF^l)^	1 m KOH	0.5 m Hydrazine	Hydrazine oxidation	0.018, 1.0	—	—	[22]
CoP/TiM^m)^	1 m KOH	0.1 m Hydrazine	Hydrazine oxidation	−0.05, 0.2	—	—	[23]
Cu_3_P/CF^n)^	1 m KOH	0.5 m Hydrazine	Hydrazine oxidation	0.152, 0.72 (100)	—	—	[24]
CuCo_2_O_4_	1 m KOH	Glycerol	Formic acid	1.38	89.1	—	[25]
Ni_2_P NPA/NF^o)^	1 m KOH	10 mm HMF	FDCA	1.35, 1.44	98	—	[26]
Ni_3_S_2_/NF	1 m KOH	10 mm HMF	FDCA	1.35, 1.46	98	—	[27]
Co–P/CF	1 m KOH	50 mm HMF	FDCA	1.38, 1.44	—	—	[28]
Co_3_O_4_	1 m KOH	HMFOR	FDCA	1.4	90.35	92.42	[29]
S—Ni@C^p)^	1 m KOH	HMF	FDCA	1.35	96	96	[30]
Nano‐Cu foam	0.1 m KOH	5 mm HMF	FDCA	1.25	95.3	—	[31]
NiB_x_—P_y_ ^q)^	0.1 m KOH	HMF	FDCA	1.464	92.5	90.6	[32]
NiO_x_/MWCNTs‐O_x_ ^r)^	1 m KOH	Glycerol	Oxalate	1.31	—	—	[33]
Ni_2_P/Ni/NF^s)^	1 m KOH	30 mm Furfural	Furoic acid	1.43, 1.48	100	—	[34]
Pd^0^ NPs	PivOH	Diphenyl ether	Dibenzofuran	—	—	72	[35]
Fe—CoP/CC^t)^	1 m KOH	5 mL of Aloe extract	—	1.572, 1.44	—	—	[36]
Pd_x_Auy/C^u)^	3 m KOH	Sorbitol	Sorbitol oxidation	−0.43	—	—	[37]
CoP NWs/CC^v)^	1 m KOH	40 mg L^−1^ Triclosan	Phenol	1.54, 1.63	—	—	[38]
Fe_2_P/SSM^w)^	10 m KOH	0.5 m Glucose	Glucose oxidation	1.33, 1.22	—	—	[39]
t‐Ni/Co MOF^x)^	1 m KOH	Benzylamine	Benzonitrile	1.30 V	73	80–94	[40]
2D cMOF^y)^	1 m KOH	Benzylamine	Benzonitrile	1.29 V	≈87	—	[41]

^a)^ 3D nanostructured TiO_2_ nanotube arrays (TNTA‐web) as a support for Pd nanoparticles (NPs)

^b)^ F‐modified *β*‐FeOOH

^c)^ Cobalt triphosphine complexes

^d)^ CuCoN_x_‐based porous nanosheet arrays grown on a carbon fiber (CF) with a conductive nitrogen‐doped carbon shell

^e)^ zirconium‐based UiO‐68‐(2,2,6,6‐tetramethylpiperidin‐N‐oxyl)

^f)^ 3D hierarchically porous nickel‐based electrocatalyst

^g)^ single‐crystalline NiS_2_ nanostructure film grown directly on a carbon fiber cloth

^h)^ octahedral ruthenium complex

^i)^ cobalt oxide (Co_3_O_4_) nanosheets on carbon paper

^j)^ CuO nanorods

^k)^ small‐sized MnO_2_ nanocrystals

^l)^ Ni_2_P nanoarray grown on nickel foam

^m)^ CoP nanarray on Ti Mesh

^n)^ Cu_3_P nanoarray on copper foam

^o)^ 3D Ni_2_P nanoparticle arrays on nickel foam

^p)^ sulfur‐modulated metallic nickel NPs coupled with carbon frameworks

^q)^ P‐doped NiB_x_

^r)^ NiO_x_ embedded on oxygen‐functionalized multiwalled carbon nanotubes

^s)^ Ni_2_P‐derived arrays on nickel foam

^t)^ Fe‐doped CoP nanosheet array

^u)^ bimetallic catalysts of palladium and gold

^v)^ CoP nanowires/carbon cloth

^w)^ Iron phosphide films grown in situ on stainless steel mesh

^x)^ bimetallic Ni/Co metal‐organic framework derivative

^y)^ 2D Conductive Metal–Organic Framework Nanowires.

### 2.1 Oxidation of Alcohols

Due to the wide applications in commodity chemical production, alcohol oxidation reactions (AOR) are considered one of the most essential anodic reactions. By electrochemical oxidation, alcohols are treated more environmentally friendly, as it produces benzyl alcohol (BA) and benzoic acid, which are used in synthetic fibers, resins, antiseptics, etc.^[^ ^42^ ^]^ Direct alcohol fuel cells (DAFC) become a significant technology to develop environmentally friendly organic synthesis processes in the past decade. The electrocatalytic oxidation of alcohols such as methanol, ethanol, and poly alcohols, which are commonly used in fuel cells and energy production, is a key area of research. To synthesize chemicals, electrochemical methods can be used to oxidize alcohols to aldehydes and acids selectively.

#### 2.1.1 Monohydric Alcohols

It is widely regarded as one of the most fundamental transformations in organic synthesis and industrial chemistry, which allows monohydric and polyhydric alcohols to undergo selective oxidation to produce carbonyl compounds.^[^ ^43^ ^]^ They are typically oxidized with stoichiometric amounts of chromium salts, oxalyl chlorides, or hypervalent ions.^[^ ^44^ ^]^ However, these processes often have serious drawbacks, including high costs for solvents and catalysts, the high toxicity of organic residues, excessive waste disposal, and potential explosions. In organic chemistry, using air or oxygen during the liquid phase of an ethanol oxidation reaction (EOR) has become a promising approach in recent years.^[^ ^45^ ^]^ Aerobic oxidation chemistry uses air as the predominant, environmentally benign, and economic oxidant. Oxygen is difficult to activate due to its triplet ground state structure. Consequently, the air or oxygen oxidation process is subjected to harsh reaction conditions, such as high temperature (T) and/or high pressure, which may even require precious metal catalysts. Due to its advantages of replacing dangerous redox reagents with electric current, reducing the generation of toxic waste, and simplifying the whole process, electrocatalytic oxidation technology has become a large‐scale application of traditional chemical processes.^[^ ^46^ ^]^ Several factors, including low operating T, high energy density, and low environmental impact, make ethanol and methanol the most promising for use as fuels.^[^ ^47^ ^]^ Although many electrocatalysts are developed, only a few are capable of oxidizing monohydric alcohols. Several strategies have been developed and implemented for synthesizing multicomponent catalysts and/or nanocomposite catalysts. It is possible to alter the catalyst surface's electronic structure and optimize the adsorbates' binding energy by alloying Pd or Pt with transition metals in 3D structures.^[^ ^48^ ^]^

Using a hydrothermal method and a combination of ammonization treatment and electrochemical deposition, Li et al. synthesized porous Co_3_N‐Ni_3_N nanowire‐supported carbon fiber cloth as a catalyst.^[^ ^49^ ^]^ Adding metallic Co_3_N‐Ni_3_N to electrocatalysts can improve poisoning tolerance without sacrificing the electrodes' good conductivity. Moreover, due to the hierarchical nanostructure of Co_3_N‐Ni_3_N, abundant active sites are exposed, and mass transfer kinetics is excellent. Under alkaline conditions, the Pd/Co_3_N‐Ni_3_N/CFC catalyst exhibits more significant activity and stability than the Pd catalyst. Recently, Weiyu et al. reported a general method for fabricating ternary ultra‐thin PtNiM (M = Rh, Os, and Ir) nanowires.^[^ ^50^ ^]^ They are highly efficient electrocatalysts for alcohol oxidation due to their adjustable composition and uniform particle size under 2 nm. PtNiRh nanomaterials have greater electrocatalytic activity and stability than PtNi nanomaterials among multi‐metal NWs for methanol oxidation reaction (MOR) and EOR. Using a simple thermal treatment method, Guoqiang et al. prepared Co_0.83_Ni_0.17_ alloy NPs on activated carbon (Co_0.83_Ni_0.17_/AC).^[^ ^51^ ^]^ In addition, Co_0.83_Ni_0.17_/AC exhibits a porous structure with a specific surface area of 159.2 m^2^ g^−1^. In electrocatalysis, Co_0.83_Ni_0.17_/AC has the following advantages: i) The porous structure with a high specific surface area improves the mass transfer and exposes the active catalytic site; ii) Co_0.83_Ni_0.17_ alloy NPs embedded in activated carbon can enhance conductivity while forming Co/Ni‐dependent active species in situ; iii) electrocatalytic oxidation of organics in alkaline media is possible with high‐valency Co/Ni active species (e.g., CoOOH, NiOOH). Toluene is almost exclusively converted to benzoic acid (pH‐COOH) via oxidation, which consumes massive energy and resources. By increasing pressure (1 MPa), T (150–170 °C), and chemical oxidants, the environment is polluted and resources are wasted.^[^ ^52^ ^]^ To alleviate resource depletion and severe environmental degradation, new environmentally friendly and sustainable ways to continuously produce Ph‐COOH are essential. These requirements can be met by electrocatalytic chemical oxidation with hydrogen production, which combines mild working conditions, low thermodynamic barriers, and energy savings. This can be met by mixed hydrolysis, which combines electrocatalytic chemical oxidation with hydrogen production, due to its gentle operation conditions, low thermodynamic barrier, and energy savings.^[^ ^42^, ^53^ ^]^

A production density of more than 350 mA cm^−2^ will result in competition between oxygen evolution reaction (OER) and electrocatalytic benzyl alcohol oxidation (EBO) if implemented today, thereby decreasing EBO's efficiency. An electrocatalyst with strong EBO activity and a slow OER profile must conduct mixed water electrolysis at high current densities. Co_3_O_4_ nanoneedle arrays had higher EBO activity than Co_3_O_4_ NPs, according to a recent study by Yin et al.^[^ ^42b^ ^]^ In the EBO electrocatalyst, Ni, Co hydroxide grows on nickel (Ni) foam, generating 400 mA cm^−2^ at just 1.42 V.^[^ ^42a^ ^]^ Huang et al. used interface engineering to create local crystallinity in a Fe/Co (oxide) heterostructure, dramatically raising its overpotential to 1.42 V when delivering 10 mA cm^−2^.^[^ ^54^ ^]^ A MOF NS with exposed bimetal active sites may be the best choice for EBOS applications due to its low dimensionality. 2D MOF electrocatalysts were developed by Song et al. for the oxidation of BA.^[^ ^55^ ^]^ 2D‐NiCo‐61‐MOF/NF has a higher electrocatalytic activity than other 2D‐MOF/NF. Compared with a reversible hydrogen electrode (RHE), 2D‐NiCo‐61‐MOF/NF requires only 1.52 V to achieve 338.16 mA cm^−2^. After 20 min of continuous electrocatalysis at 1.42 V (vs RHE), NiCo‐61‐MOF/NF had a current density of 38.67 mA cm^−2^ and a retention rate of 77.34%. The electrocatalytic kinetics and density functional theory (DFT) experiments suggest that NiCo‐61‐MOF/NF has an ultra‐thin 2D structure, more exposed active centers, and a more realistic electronic structure toward BA molecules, which allows EBO to represent the reaction more realistically.

**Figure 1:**
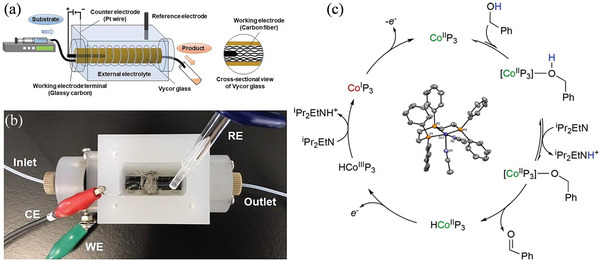
a) Schematic illustration of a column flow cell for electrochemical reaction. b) Top view. Reproduced with permission.^[^ ^11^ ^]^ Copyright 1969, Elsevier. c) BA oxidation on Co triphosphene complex. Reproduced with permission.^[^ ^12^ ^]^ Copyright 2021, American Chemical Society.

Suga et al. demonstrated the electrochemical oxidation of alcohols using a column flow cell (**Figure** **1**a,b).^[^ ^11^ ^]^ The voltammetric measurement shows that 1‐phenylethanol and benzaldehyde are more easily oxidized in the presence of an Au electrode and an alkaline media. The researchers modified carbon‐fiber thread with Au NPs to perform such a reaction in a column flow cell. A modified column carbon‐fiber thread electrode easily oxidizes benzylic, allylic, and aliphatic alcohols with hydroxy groups with Au NPs, which have a high surface area. Heins et al. showed that the tridentate ligand bis(2‐diphenylphosphinoethyl)phenylphosphine (P_3_) coordinates with the cobalt forms [(CH_3_CN)_2_Co^2+^P_3_](BF_4_)_2_ (Co^2+^P_3_).^[^ ^12^ ^]^ At an applied potential of −630 mV versus Ferrocenium/Ferrocene (Fc^+/0^), Co^2+^P_3_ electrocatalytically oxidizes BA to benzaldehyde in the presence of the Brönsted base iPr_2_EtN and obtains a turnover frequency (TOF) of 19.9. Co^2+^P_3_ is reduced by one electron in the presence of excess BA, and iPr_2_EtN to [(CH_3_CN)_2_Co^1+^P_3_]BF_4_ (Co^1+^P_3_) and half an equivalent of benzaldehyde is formed simultaneously. BA undergoes stoichiometric oxidation, indicating electron transfer between intermediate Co species and the starting species Co^2+^P_3_. The kinetics and computational studies show an unfavorable pre‐equilibrium step followed by a favorable deprotonation step. Based on kinetic and theoretical studies, Figure 1c illustrates a proposed catalytic cycle for BA oxidation, which begins with BA coordinating with Co^2+^P_3_. Alcohol binding is unfavorable based on saturation kinetics, the absence of alcohol adducts, and computation results. An alkoxide complex is formed by deprotonating coordinated alcohol. A reaction sequence based on computations, base dependence, and BnOLi reactivity is supported. Calculations indicate that the bound alkoxide can be eliminated by *β*‐hydride, forming aldehyde and HCo^2+^P_3_. In stoichiometric and catalytic pathways, HCO^2+^P_3_ represents a point of divergence. A catalytic reaction occurs when [FeCp*_2_]^+^ or an electrode oxidizes the HCo^2+^P_3_; a stoichiometric reaction occurs when Co^2+^P_3_ and HCo^2+^P_3_ transfer electrons to one another. Both scenarios lead to the formation of HCo^3+^P_3_ complexes that can be readily deprotonated to produce Co^1+^P_3_. Last, the cycle is closed by the oxidation of Co^1+^P_3_ to Co^2+^P_3_.

**Figure 2:**
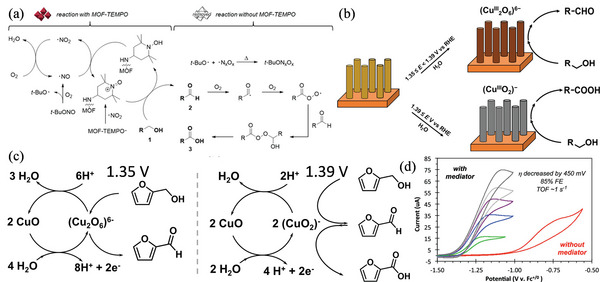
a) Oxidation reaction control by MOF–TEMPO. Reproduced with permission.^[^ ^14^ ^]^ Copyright 2020, American Chemical Society. b) Electrooxidation of alcohols to aldehydes using CuO nanorods (NR) in aqueous electrolyte. c) Electro‐catalytic oxidation of FA on CuO NR. Reproduced with permission.^[^ ^20^ ^]^ Copyright 2021, American Chemical Society. d) Alcohol oxidation on regenerative H atom accepter (mediator). Reproduced with permission.^[^ ^17^ ^]^ Copyright 2020, American Chemical Society.

Kim et al. devised a robust, efficient approach for progressively oxidizing alcohols to carboxylic acids using a highly functionalized heterogeneous MOF.^[^ ^14^ ^]^ By oxidizing alcohols to carboxylic acids with broad functional group tolerance, such as 2,5‐furandicarboxylic acid and 1,4‐benzenedicarboxylic acid, the MOF in‐and‐out approach may also be employed to generate carboxylic acids of industrial and commercial importance with excellent yields and reusability. Furthermore, MOF‐2,2,6,6‐tetramethylpiperidin‐N‐oxyl (TEMPO) functions as an antioxidant stabilizer, avoiding undesired aldehyde oxidation, and the flawless recovery capabilities of such a MOF necessitates a reconsideration of MOFs' benefits as catalysts and in related domains. **Figure** **2**a depicts a representation of the consecutive one‐pot oxidation processes. The presence of MOF‐TEMPO is crucial to achieving such oxidation processes. After treatment with tert‐butyl nitrite compounds, the second phase of heat treatment did not affect autoxidation. The oxidation cycle of the external oxidant and the number cycle involving O_2_ and water are the two cooperative redox cycles that MOF‐TEMPO uses to convert alcohols to aldehydes in the first phase. When MOF–TEMPO is removed, the residual tert‐butyl nitrite decomposes at 80 °C or forms an inactive adduct, making the following step easier because no radicals are left behind. Fang et al. investigated the electrocatalytic oxidation of furfuryl alcohol (FA) using CuO nanorods. (Figure 2b).^[^ ^20^ ^]^ Upon examination of the surface of the working electrode, two kinds of Cu^2+^ intermediates are detected, namely (CuO_2_)^−^ and (Cu_2_O_6_)^6−^. The (Cu_2_O_6_)^6−^ induces conversion of FA to furaldehyde, and produces a yield of ≥98% with a potential range of 1.35–1.39 V. The amount of 2‐furoic acid obtained was ≥99% when charging (CuO_2_)^−^ above 1.39 V (vs RHE). Cu^3+^‐catalyzed systems show a degree of universal applicability due to their ability to convert BA, vanillyl alcohol, and 4‐pyridinemethanol into aldehydes and acids, respectively. According to the above results, oxygen is transferred in two steps during the electrocatalytic oxidation of FAs in an aqueous solution using the CuO‐NR catalyst (Figure 2c). At the potential region between 1.35 and 1.39 V versus RHE, CuO is electro‐oxidized to (Cu_2_O_6_)^6−^ using CuO + 4H_2_O → (Cu_2_O_6_)^6−^ + 8H^+^ + 2e^−^ or is electro‐oxidized to (Cu^3+^O_2_)^−^ using CuO + H_2_O → (CuO_2_)^−^ + 2H^+^ + e^−^ at a potential greater than 1.39 V (vs RHE). CuO is generated by the reaction between Cu^3+^ species and FA and/or furaldehyde. As evidenced by the isotope tracer technique, the ^18^O atoms from H_2_^18^O were transferred to the organic product via the CuO‐NR catalyst. McLoughlin et al. proposed that the ruthenium octahedral complex [Ru(acac)_2_(pyimN)] (Ru^3+^N_3_) might be exploited as a potential electrochemically regenerable hydrogen atom acceptor in tandem electrocatalytic reactions to reduce the overpotential for electrocatalytic alcohol oxidation by 450 mV (Figure 2d).^[^ ^17^ ^]^ A catalyst capable of oxidizing 2‐propanol to acetone at 0.70 V over ferrocene/ferrocene (Fc^+/0^) is XNN(dppb)[1,X = Cl, 2,X = H] on Fc^+/0^. With a TOF of ca. 1s^−1^ in tetrahydrofuran, acetone is electrocatalytically oxidized by RuCl upon the addition of Ru^3+^NH_4_. A cyclic voltammetry (CV) experiment and a chemical hydride atom transfer experiment indicate that RuH_2_ is primarily electrochemically reduced to Ru^3+^N_3_ (Figure 2d).

Li et al. created a novel catalyst consisting of Au NPs and cobalt oxyhydroxide NSs (Au/CoOOH) that enhanced the use of hydrogen in AORs at high current densities.^[^ ^56^ ^]^ In the presence of 1 m KOH and 0.1 m BA, Au/CoOOH has a current density of 340 mA cm^−2^ at 1.3 V versus RHE at room temperature. BA oxidation and hydrogen generation rates are 26‐ and 28‐fold higher than Au at 1.3 V versus RHE, respectively. Current density can be increased to 540 mA cm^−2^ at 1.5 V versus RHE, the highest value reported so far at such a low potential. An electrolyzer with two electrodes and no membrane can reach 4.8 A at 2.0 V, suggesting that this catalyst could be employed in industrial settings. Based on experiments and spin polarization DFT, BA (in the form of alkoxide) accumulates at the Au/CoOOH interface and is oxidized by electrophilic OH^*^ produced on CoOOH. The activity is more significant than pure Au due to the low reaction barrier. The current density of an Au/CoOOH is 9–28 times that of Au, and it can handle alcohols with *α*‐bonds, such as phenyl, C=C, and C=O groups. Anodic potential/open circuit facilitates reversible oxidation/reduction of catalysts in long‐term electrooxidation.^[^ ^56^ ^]^

#### 2.1.2 Polyhydric Alcohols

**Figure 3:**
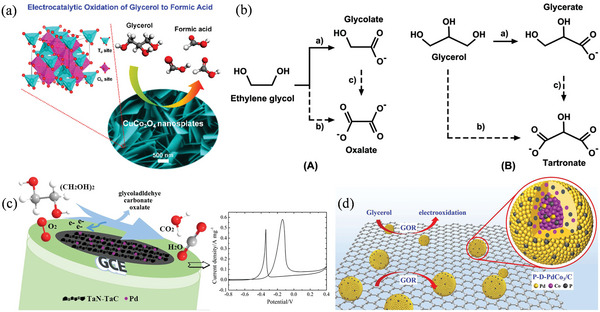
a) eGOR on CuCo_2_O electrocatalyst. Reproduced with permission.^[^ ^25^ ^]^ Copyright 2020, American Chemical Society. b) Schematic representation of A) ethylene glycol and B) eGOR at AuPNR^6‐50^ aerogels. Reproduced with permission.^[^ ^64^ ^]^ Copyright 2020, American Chemical Society. c) Oxidation of ethylene glycol on Pd‐TaN‐TaC electrocatalyst. Reproduced with permission.^[^ ^65^ ^]^ Copyright 2020, Elsevier. d) eGOR on Pd‐PdCo_3_ NPs. Reproduced with permission.^[^ ^66^ ^]^ Copyright 2021, Elsevier.

The catalytic conversion of polyols (e.g., diols, triols, sugars, cyclohexane, and cellulose) into high value‐added chemicals (e.g., polyurethane, polyester, polycarbonate) holds the potential applications in the chemical industry, and electrocatalysis has received more attention as well.^[^ ^57^ ^]^ Due to the indisputable advantages of polyols, glycerol, and ethylene glycol, such as their high boiling point, low toxicity, and low volatility, direct polyol fuel cells have attracted worldwide interest.^[^ ^58^ ^]^ Till now, pure platinum catalysts have been recognized as good anode catalysts for oxidizing alcohol. A substantial hurdle to DAFCs' development is the scarcity, high cost, and limited activity of platinum resources on the earth.^[^ ^59^ ^]^ Thus, many efforts are focused on designing and developing high‐performance non‐noble metal catalysts for fuel cell oxidation reactions. Diol oxidative cleavage has been studied electrochemically in which sodium periodate is generated anodically in a biphasic system,^[^ ^60^ ^]^ and unmediated diol cleavage has also been observed.^[^ ^61^ ^]^ As the mediator, Et_4_NBr was used by Onomura and colleagues to efficiently oxidize 1,2‐diols into ‐hydroxyketones.^[^ ^62^ ^]^ Me_2_SnCl_2_ is formed by the reversible breakdown of the SnO link in vicinal diol, producing stannylene acetal through halide‐assisted alcohol oxidation. The galvanostatic protocol only requires a catalytic quantity of the organotin reagent. On the other hand, the analogous chemical approach requires the preformation of the stannylene acetal in a separate step. Anodic oxidation of the secondary alcohol is observed to occur preferentially in 1,2‐diols containing both primary and secondary alcohol groups. The reaction method also effectively distinguishes 1,2‐diols from 1,3‐diols or isolated hydroxyl groups. The organotin reagent used in this reaction can be replaced with a copper salt, and the addition of a chiral ligand allows for the asymmetric electrochemical oxidation of 1,2‐diols, amino alcohols, and amino aldehydes into *α*‐hydroxyketone or *α*‐aminoesters with significant enantioselectivity.^[^ ^63^ ^]^ Electrochemically oxidizing glycerol can produce high‐value compounds like formic acid, which can then be utilized in indirect or direct formic acid fuel cells. Han et al. investigated cobalt‐based spinel oxide nanostructures (MCo_2_O_4_, M = Mn, Fe, Co, Ni, Cu, and Zn) as reliable electrocatalysts for the selective electricatalytic oxidation of glycerol (eGOR) to produce formic acid, (**Figure** **3**a).^[^ ^25^ ^]^ In alkaline electrolytes, CuCo_2_O_4_ > NiCo_2_O_4_ > CoCo_2_O_4_ > FeCo_2_O_4_ > ZnCo_2_O_4_ > MnCo_2_O_4_ is the sequence of their intrinsic catalytic activity. With a constant potential of 1.30 V (vs RHE), an 80.6% selectivity for formic acid production is achieved, as a Faradaic efficiency (FE) of 89.1% for all value‐added products and a 79.7% conversion rate for glycerol. Several structural characterization approaches have been used to determine the stability of the CuCo_2_O_4_ catalyst. These discoveries offer the possibility of producing formic acid at low temperatures using earth‐abundant electrocatalysts from glycerol. Au_PNR_^6–50^ aerogels with varying percentages of [110] faces (from 12 to 36%) were prepared and used as the catalyst in the ethylene glycol oxidation reaction.^[^ ^64^ ^]^ The specific activity and long‐term stability are significantly influenced by the percentage of the [100] and [111] components added together as well as the fraction of the [110] factors. It has been demonstrated that the most stable Au_24_^6–50^ aerogels possess over 95% of their original current after several years while also possessing a high specific activity (about 90.42 mA cm^−2^). A five‐membered ring structure, made up of two Au atoms and two oxygen atoms, would form on the surface of Au‐based catalysts. Glycerate or glycolate is the intermediate product of the electrooxidation of ethylene glycol or glycerol on Au‐based catalysts in an alkaline solution. Mechanism study shows that each major intermediate contains a hydroxyl group (Figure 3b), except for a carboxylate group. In the presence of these intermediates, a six‐membered ring structure forms, consisting of two Au atoms with two oxygen atoms separated from the carboxylate and hydroxyl groups (referred to as a six‐membered ring structure). Moreover, each minor stage in the oxidation of small molecules, that is, polyhydric alcohols (such as oxalate and tartronate) has two carboxylate groups. Li et al. reported Pd NPs deposited on TaN‐TaC composites^[^ ^65^ ^]^ as an advanced fuel cell electrode (Figure 3c). High catalytic activity is observed for ethylene glycol electrooxidation on Pd/TaN‐TaC electrocatalyst‐modified glassy carbon electrode in an alkaline medium. Using a facile dealloying and phosphating process, Imhanria et al. presented a phosphated dealloyed PdCo_3_ (P‐D‐PdCo_3_/C) composite catalyst to investigate eGOR (Figure 3d).^[^ ^66^ ^]^ In the composite of P‐D‐PdCo_3_, Pd, Co, and P in the de‐alloyed and phosphated form have good Pd utilization and synergy, showing a good electrocatalytic activity of 65.9 mA mg^−1^ and more than 3000 s of stability in eGOR.

**Figure 4:**
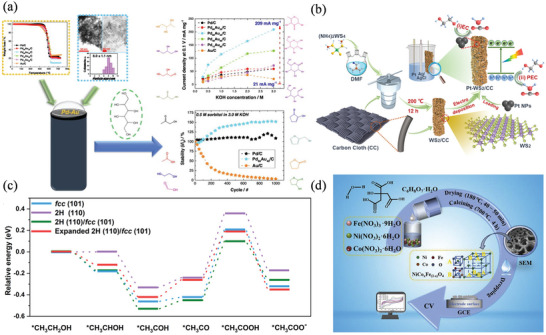
a) SOR on Pd_x_Au_y_/C catalyst. Reproduced with permission.^[^ ^37^ ^]^ Copyright 2021, Elsevier. b) Electro‐oxidation of ethylene glycol on Photoactivated Pt anchored 3D WS_2_‐CC. Reproduced with permission.^[^ ^69^ ^]^ Copyright 2020, American Chemical Society. c) Free energies of electro‐catalytic EOR on different surfaces. Reproduced with permission.^[^ ^70^ ^]^ Copyright 2021, American Chemical Society. d) eGOR on Co‐doped Ni—Fe spinels. Reproduced with permission.^[^ ^71^ ^]^ Copyright 2022, Elsevier.

NiFeO_x_ and NiFeN_x_ were synthesized from NiFe‐LDH NS arrays toward glucose anodic oxidation and HER cathodic reduction, respectively.^[^ ^67^ ^]^ a current density of 200 mA cm^−2^ at 1.48 V is reached, which surpasses most of the transition metal‐based electrodes to date. Gao et al. used a simple and universal method to prepare 3D Pd—M (M = Ag, Pb, Au, Ga, Cu, and Pt) NSs.^[^ ^68^ ^]^ This 3D structure provides a high specific surface area, which is beneficial for enhancing catalysis. Results show that Pd‐M‐NSs is highly catalytic and stable for EOR, ethylene glycol oxidation, and MOR. Among the optimized Pd_7_Ag NSs, 7.08 A mg^−1^/14.3 mA cm^−2^ shows the greatest mass‐to‐specific activity, 7.01 A mg^−1^/14.1 mA cm^−2^ for eGOR, and 2.18 A mg^−1^/4.4 mA cm^−2^ for MOR, outperforming the Pd/C catalysts. Torres‐Pacheco et al. investigated the sorbitol electrooxidation reaction (SOR) employing bimetallic catalysts of Pd_x_Au_y_/C, as illustrated in **Figure** **4**a.^[^ ^37^ ^]^ At a fixed potential of 0.1 V, Pd_40_Au_60_/C and Pd_60_Au_40_/C show the best activities when their onset potentials are 0.43 V versus NHE, and the current densities are 128 and 209 mA cm^−2^, respectively. The high SOR activity is mainly ascribed to the bimetallic interactions, which change the lattice parameters and the binding energies of Pd. Recently, Pt NPs electrodeposited onto WS_2_/CC exhibited a high electrocatalytic activity (9931 mA mgPt^−1^) toward ethylene glycol, which outperforms Pt/CC (2050 mA mgPt^−1^) and commercial Pt/C catalysts (575 mA mgPt^−1^) by 4.84 and 17.3 times, respectively (Figure 4b).^[^ ^69^ ^]^ Au@Pd core‐shell nanorods with a distinctive fcc‐2H‐fcc heterophase were prepared using a wet‐chemical synthesis approach, and exhibited a mass activity of 6.82 A mgPd^−1^ for EOR.^[^ ^70^ ^]^ This value also outperforms commercial Pd/C, 2H‐Pd NPs, and fcc‐Pd NPs. Based on DFT calculations, the increased performance of the heterophase Au@Pd nanorods is due to the lattice expansion of the Pd shell and the border of the 2H/fcc phase, which lowers the energy barrier for intermediate formation (Figure 4c). Using the sol‐gel method, Zhong et al. prepared a series of Co‐doped NiFe_2_O_4_, which were used as efficient electrocatalysts for eGOR (Figure 4d).^[^ ^71^ ^]^ Due to the uneven oxygen distribution caused by the Co doping, oxygen defect sites are formed in NiFe_2_O_4_, which enhances the electrocatalytic properties.

Li et al. reported the simultaneous production of hydrogen and value‐added formate from aqueous glycerol solutions using Ni—Mo—N/CFC.^[^ ^72^ ^]^ A cathode‐catalyzed Ni—Mo—N/CFC reaction converts electron‐reduced water into hydrogen, whereas an anode‐catalyzed Ni—Mo—N/CFC reaction transforms glycerol into formate, a more valuable product than OER. The electrocatalytic performance of NiMoN/CFC on the anodic eGOR exhibits a moderate OER activity at the potential of 1.57 V versus RHE. In addition, the anode potential at 10 mA cm^−2^ decreases significantly to 1.30 V after adding 0.1 mg of glycerol to the solution. Mechanism study reveals that the concentrations of formate increased and the glycerol concentrations decreases over time, indicating that glycerol is effectively converted into formate. Finally, the glycerol has been fully converted to formate with a 93% yield.

### 2.2 Oxidation of Aldehydes

**Figure 5:**
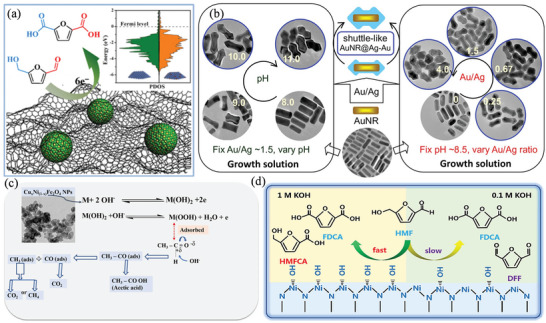
a) Electrooxidation of HMF on S‐Ni@C electrocatalyst. Reproduced with permission.^[^ ^30^ ^]^ Copyright 2021, American Chemical Society. b) Oxidation of formaldehyde on AuNR@Ag nanostructures. Reproduced with permission.^[^ ^84^ ^]^ Copyright 2020, Elsevier. c) Mechanism of acetaldehyde oxidation on CuNFNPs. Reproduced with permission.^[^ ^85^ ^]^ Copyright 2020, The Author(s). d) Biomass oxidation over Ni‐nitride. Reproduced with permission.^[^ ^86^ ^]^ Copyright 2021, Chinese Academy of Sciences. Published by Elsevier and Science Press.

Oxidation is convenient to convert aldehydes into carboxylic acids and high‐value‐added chemicals.^[^ ^73^ ^]^ It is also common for converting carbohydrates, sugars, and other biomass raw materials into value‐added products.^[^ ^74^ ^]^ The use of electrocatalysts in oxidation reactions is becoming more popular, particularly for synthesizing complex molecules, such as natural products and drugs.^[^ ^75^ ^]^ Aldehyde is toxic and cannot be used with fuel cells. Aldehyde oxidation, by contrast, is used in textiles and electroless copper plating. As part of oxidizing formaldehyde in 0.1 m NaOH solution by partial galvanic replacement, Raoof, Aghajani, and colleagues formed bimetallic PdCu particles on the surface of carbon nanotube paste electrodes.^[^ ^76^ ^]^ Through N‐heterocyclic carbene (NHC) catalysis, Boydston developed a method to convert aldehydes into esters electrochemically.^[^ ^77^ ^]^ At low potential (+0.1 V vs Ag/AgNO_3_) Breslow intermediates form between aldehydes and NHCs, which are oxidized to ester intermediates when intercepted by alcohols. Electrocatalysts based on precious noble metals, such as Pd, Pt, Au, or their bimetal alloys, are employed for electrocatalytic oxidation reactions.^[^ ^78^ ^]^ Recently, researchers have focused on developing earth‐abundant, low‐cost electrocatalysts that are selective and active for these oxidation reactions. Miao et al. reported energy‐saving hydrogen production using Co_3_FeP_x_@NF and glucose, fructose, maltose, and sucrose.^[^ ^79^ ^]^ However, a voltage of 1.35 V is required to achieve an electrical density of 10 mA cm^−2^. Liu et al. demonstrated an anodic glucose oxidation reaction in combination with the cathodic HER by using nanostructured NiFeO_x_‐NF and NiFeN_x_‐NF.^[^ ^67^ ^]^ A higher potential and current density are observed for both NiFeOx‐NF and NiFeNx‐NF for glucose oxidation reaction and HER, respectively. NiFeO_x_‐NF electrodes are used as anode and cathode to produce gluconic acid and hydrogen gas at a voltage of 1.48 V. The electrochemical cleavage of C_a_—C_b_ bonds in lignin models using tert‐butyl hydroperoxide (t‐BuOOH) has also been reported to produce aromatic aldehydes and phenols.^[^ ^80^ ^]^ The electron‐generated radicals of C_b_ from substrates and the electrons generated by t‐BuOOH readily undergo cross‐coupling reactions, dissolving the C_a_—C_b_ bond. This strategy allows the synthesis of aromatic aldehydes from many lignin model dimers, polymers, and even macromolecular lignin. For complex biomass electrolysis, inorganic mediators have also been shown to be useful, such as polyoxometalates^[^ ^81^ ^]^ and metal ions,^[^ ^82^ ^]^ resulting in reduced energy consumption (B 83%) and the creation of organic oxygenates (e.g., formic acid).^[^ ^81^ ^]^ A wide range of chemical synthesis applications is achieved by electrocatalytic oxidation of hydroxymethylfurfural (HMF), producing 2,5‐furandicarboxylic acid (FDCA), 5‐formyl‐2‐furancarboxylic acid (FFCA), and 5‐hydroxymethyl‐2‐furancarboxylic acid (HFCA).^[^ ^83^ ^]^ By combining sulfur‐modified metallic Ni NPs and carbon frameworks (S‐Ni@C) via two steps, a high FE of 96%, nearly 100% conversion, and a 96% yield of HMF are achieved (**Figure** **5**a).^[^ ^30^ ^]^ Using gold nanorod/silver (AuNR@Ag) nanostructures, Li et al. investigated the factors controlling shuttle‐like shapes, showing that the residual Au precursor is crucial to the formation (Figure 5b).^[^ ^84^ ^]^ The peaks in the forward scan most likely reflect electrochemical oxidation catalyzed by monometallic Ag or Au atoms, and a low‐potential peak near −0.1 V reflects an oxidation reaction catalyzed by adjacent Ag and Au atoms. Khalaf et al. prepared Cu_x_Ni_(1−x)_Fe_2_O_4_ NPs (CuNFNPs) in KOH electrolytes using combustion and calcination, which were used as the catalysts for the AOR in an alkaline medium (Figure 5c).^[^ ^85^ ^]^ CuNFNPs are applied as a physical substrate in KOH‐medium and activated via CV to produce MOOH, a metal hydroxide with a polar bond. Therefore, the activated CuNFNPs provide an appropriate surface for the adsorption of acetaldehyde that has C=O. Using the CuNFNPs as a catalyst, acetaldehyde can then be converted to CH_3_CO (adsorbed on the surface of CuNFNPs) by breaking the bond between C—H in the CHO group. Adsorption of CH_3_CO to OH media can result in the conversion of CH_3_CO to CO_2_ or CH_4_. By poisoning the catalyst with products like acetic acid and/or CO_2_, CuNFNPs may lose their electroactivity. Zhou et al. demonstrated that Ni_3_N exhibits good electrocatalytic activity for the electrochemical oxidation of HMF (HMFOR) in the alkaline electrolyte.^[^ ^86^ ^]^ During this process, Ni_3_N undergoes a two‐step reaction during which Ni atoms lose electrons and adsorb OH, resulting in Ni^2+*δ*^N(OH)_ads_ and facilitating the HMFOR process due to the increased active species (Figure 5d).

Yang et al. reviewed the detailed recent developments in HMFOR, including the advantages and disadvantages of noble metals catalysts, non‐noble metals, and non‐metals.^[^ ^78^ ^]^ Carbon numbers (r6) for biomass derivatives are typically low. In oxidative outer‐sphere reactions, organic substrate molecules can undergo single electron transfer (SET) into radical cations. By linking these chemical radicals to each other through Kolbe electrolysis^[^ ^87^ ^]^ or Michael addition (a reaction between any Michael donor (*α*,*α* disubstituted carbonyls, nitrile, sulfone, etc.,—electron‐withdrawing groups) and any Michael acceptor (*α*,*β*‐unsaturated carbonyls, nitrile, nitro, etc.,—electron‐withdrawing groups),^[^ ^88^ ^]^ multi‐carbon compounds can be synthesized long‐term from biomass. Levulinic acid (LA) is produced from biomass when carbohydrates are transformed with acid.^[^ ^89^ ^]^ The electro‐reduction of LA has been described in the literature,^[^ ^90^ ^]^ but it never found any practical applications.^[^ ^87a^ ^]^ Using tandem electro‐reduction and Kolbe electrolysis, Schroeder and coworkers demonstrated liquid fuel production (n‐octane) from LA.^[^ ^87a^ ^]^ Anodic oxidation of carboxylic acids produces alkyl radicals for parallel C—C coupling during radical decarboxylation. Faraday and Kolbe established this in the late 1800s.^[^ ^87b^ ^]^ Using electrochemistry, Chen and colleagues transformed furan derivatives into a precursor for transportation fuel.^[^ ^88^ ^]^ From the reaction of 2‐methylfuran with graphite electrode cation radicals, 3‐(5‐methylfuran‐2‐yl)hexane‐2,5‐dione, a derivative of 2,5‐dimethylfuran, has been obtained. With further hydrodeoxygenation, it is possible to obtain a branched alkane fuel with a 91% yield by hydrodeoxygenating 4‐ethylnonane. Furoic acid is now made from furfural using a disproportionation reaction in a concentrated alkaline solution, although the theoretical yield is rigorously limited to 50% due to a stoichiometric quantity of furfural alcohol as a by‐product.^[^ ^91^ ^]^ Despite the fact that the catalytic aerobic oxidation of furfural produces measurable quantities of furoic acid, the use of noble metal catalysts and severe reaction conditions, such as high T and gaseous oxygen, are linked with significant capital expenditures and safety problems.^[^ ^92^ ^]^ Water as an oxidant and electricity as an energy source can be used to perform electrochemical oxidation of furfural under safe circumstances.^[^ ^34^, ^93^ ^]^ The electrochemical oxidation of furfural can also be combined with HER, which is capable of simultaneously improving biomass quality and generating hydrogen.^[^ ^34^ ^]^ Hydrogen is produced in conventional electrocatalytic systems through the cathodic HER reaction and the anodic furfural oxidation. In an alkaline electrolyte, furoic acid (or furoate) is produced as a salt of furoic acid, while the cathode produces hydrogen. Anodic furfural electrooxidation operates at a higher voltage level than cathodic furfural electrooxidation (e.g., 1.0 vs RHE), so that total voltage inputs of >1 V are usually needed to increase linked reactions. Sun's group produced furoic acid using a bifunctional array of Ni foam derived from Ni_2_P.^[^ ^34^ ^]^ Furfural oxidation onset potential is approximately 1.3 versus RHE on the anode and about 0.1 V versus RHE on the cathode, resulting in an average voltage of about 1.4 V on the integrated electrolyzer. In particular, selective electrooxidation of an organic compound can be used to reduce electricity usage and produce useful compounds at the same time by substituting anodic OER in water electrolysis.^[^ ^28^, ^39^, ^94^ ^]^ Due to inherent limitations, most of these systems operate at higher voltages than electrolysis output voltages (e.g., >1.23 V).^[^ ^95^ ^]^ A new electrocatalytic system capable of producing hydrogen and upgrading biomass more efficiently is therefore needed. Wang et al. converted electrocatalytic furfural oxidation and hydrogen production from a power input process to a power output process by coupling anodic low‐potential furfural oxidation with cathodic oxygen reduction.^[^ ^96^ ^]^ The H atom of the aldehyde group in typical furfural electrooxidation at high potentials (>1.0 vs RHE) is oxidized to H_2_O, unlike the H atom from low‐potential furfural electrooxidation at around 0 versus RHE, liberated as H_2_ gas after the breakage of C—H bond. This new electrocatalytic system can produce hydrogen and furoic acid simultaneously at the anode, obviously different from the traditional mechanism. In contrast to conventional electrolyzers, this system generates H_2_ rather than requiring electricity input, making it more interesting.

**Figure 6:**
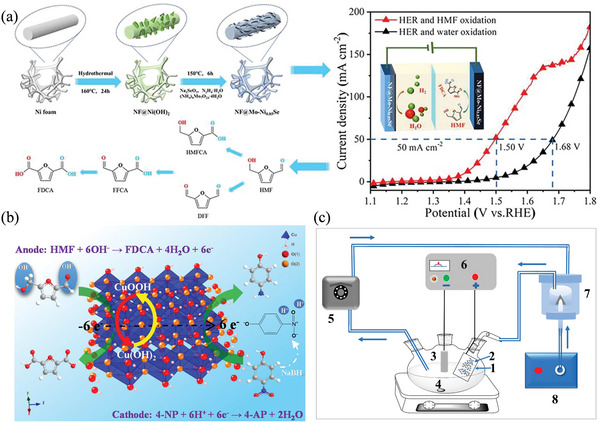
a) HMF oxidation on Mo‐Ni_0.85_Se. Reproduced with permission.^[^ ^97^ ^]^ Copyright 2021, Elsevier. b) HMF oxidation on CF‐Cu(OH)_2_ electrocatalyst. Reproduced with permission.^[^ ^98^ ^]^ Copyright 2022, American Chemical Society. c) Droplet‐assisted reactor: 1) CoP@NiF anode, 2) droplet formation, 3) Pt cathode, 4) magnetic stirrer, 5) peristaltic pump, 6) DC power supply, 7) nebulizer junction, and 8) air pump. Reproduced under the terms of the Creative Commons CC‐BY license.^[^ ^99^ ^]^ Copyright 2022, The Authors. Published by MDPI.

Yang et al. developed a bifunctional catalyst (NF@Mo‐Ni_0.85_Se) based on adding Mo‐doped Ni_0.85_Se to the Ni foam to improve the simultaneous conversion of 5‐HMF and FDCA (**Figure** **6**a).^[^ ^97^ ^]^ Molecular doping enhances electron transmission in NFN@Ni_0.85_Se and downshifts Ni's d‐band center, promoting not only the HER but also the organic hydrogen adsorption process, according to experimental electrochemical impedance spectroscopy and theoretical calculations. In basic electrolytes, catalyst pair NF@Mo‐Ni_0.85_Se is used for HER and HMF oxidation, and only a potential of 1.50 V is required to achieve a current density of 50 mA cm^−2^, which is less than total water splitting (1.68 V). Cu foam decorated with Cu(OH)_2_ (CF‐Cu(OH)_2_) is effectively used as a catalyst, and CuOOH is demonstrated to be the active species for HMFOR (Figure 6b).^[^ ^98^ ^]^ A current density of 198.2 mA cm^−2^ and an FDCA synthesis efficiency of approximately 100% (yield: 98.7%) are obtained. BA, 2‐phenoxyethanol, and HMF were electrocatalytically oxidized at ‐room temperature undergoing a droplet flow‐assisted mechanism as reported by Suliman et al.^[^ ^99^ ^]^ As a result of the combination of droplet flow and continuous flow electrochemical oxidation, aldehyde can be produced from substrate conversion at 1.3 V under ambient conditions with 97.0% selectivity using CoP as the catalyst (Figure 6c).

**Figure 7:**
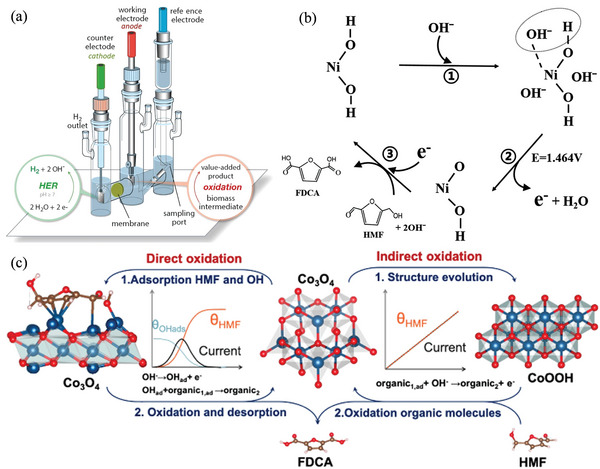
a) Schematic of a prototype electrolyzer for biomass oxidation. Reproduced with permission.^[^ ^102^ ^]^ Copyright 2021, American Chemical Society. b) Schematic process of Ni(OH)_2_ → NiOOH → Ni(OH)_2_ during the electrocatalytic oxidation of HMF. Reproduced with permission.^[^ ^32^ ^]^ Copyright 2020, American Chemical Society. c) Electro‐catalytic oxidation of aldehydes and OH group in HMF. Reproduced with permission.^[^ ^29^ ^]^ Copyright 2022, American Chemical Society.

By overgrowing surfactant‐assisted template AuNRs, Li et al. created AuNR@Ag with nanostructures with rod‐like or boat‐like forms.^[^ ^84^ ^]^ The Ag shell is grown on unpurified AuNRs with glycine added in the second step, resulting in an AuNR@Ag nanostructure with dumbbell‐like or shuttle‐like forms and the ratio of Au to Ag in the shell development solution can be adjusted easily.^[^ ^100^ ^]^ The catalyst AuNR@Ag‐Au nanostructures exhibit excellent electrochemical performances toward HMFOR. Recently, Liu et al. demonstrated that NiFe LDH NSs can be used as effective and robust anodic electrocatalysts for oxidizing HMF to FDCA.^[^ ^101^ ^]^ Because HMF oxidation has lower kinetic energy than water oxidation, it might be employed as a counter‐reaction to H_2_ evolution in high‐value organic compound‐producing water‐splitting cells. The NiFe‐LDH electrode shows an onset potential of 1.37 V (vs RHE) in HMF‐free electrolyte and achieves 20 mA cm^−2^ at a potential of 1.53 V. As a comparison, HMFOR occurs at a lower onset potential of 1.25 V, and obtains a current density of 20 mA cm^−2^ at a potential of 1.32 V. **Figure** **7**a depicts a simplified diagram of the typical setup for biomass oxidation.^[^ ^102^ ^]^ Song et al. employed a straightforward approach to produce NiB_x_ with various quantities of phosphorus, then electrocatalytically oxidized HMF.^[^ ^32^ ^]^ The FDCA yield improves first, then drops as the phosphorus level increases. When NiB_x_—P_0.07_ (nP/nNi = 0.07) is utilized as the electrocatalyst, the FDCA yield and FE are 92.5 and 90.6%, respectively (vs RHE). Figure 7b displays the electrocatalytic process of HMFOR: NiOOH receives electrons during HMF oxidation and is reduced to Ni(OH)_2_ in a heterogeneous chemical process. Lu et al. identified the direct and synergistic oxidation processes for HMFOR using Co_3_O_4_ as the catalyst.^[^ ^29^ ^]^ In the HMFOR process, Co_3_O_4_ shows a higher hydroxyl oxidation reaction barrier than the aldehyde group, due to the lower reaction barrier in the hydroxyl oxidation process (Figure 7c). NiO exhibits a high hydroxyl activity after examining the hydroxyl oxidation behaviors in transition metal oxides due to the high adsorption energy of ‐OH, which is crucial for alcohol dehydrogenation. Thus, Ni is added to improve the hydroxyl activity and then enhance the HMFOR performance. Benefiting from the dual catalytic sites, HMFOR achieves 92.4% FDCA yield and 90.35% FE.

Traditional heterogeneous catalysis has two problems. First, O_2_ and formaldehyde do not need to come into contact with Pt, because both electrochemical reactions are linked electrically so that they can be separated spatially. Second, the surface reaction is no longer limited due to adsorption configuration. A thin layer of nanoscale water‐electrolyte can be used to enhance the catalytic activity of Pt in the oxidation of formaldehyde since it establishes a physical barrier between the gas‐phase reactants and the solid‐phase catalyst.^[^ ^103^ ^]^ By taking advantage of the concentration gradient, O_2_ and formaldehyde dissolve on the surface of Pt and then diffuse toward it. Consequently, the electrolyte layer must be kept as thin as possible, otherwise, the kinetics of reaction is inhibited due to the depletion of reactants with low solubility. Besides the thin layer of n electrolyte, gas diffusion electrodes (GDEs) can solve this problem by constructing 3‐phase contact lines near catalysts, which reduce diffusion distances between the gas phase and catalyst.

### 2.3 Oxidation of Amines

**Figure 8:**
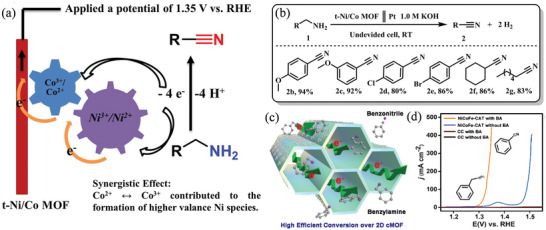
a,b) Primary amines oxidation over t‐Ni/Co MOF. Reproduced with permission.^[^ ^40^ ^]^ Copyright 2022, Elsevier. c) Oxidation of benzyl amines on 2D cMOF nanowires. d) LSV curves of BA electrooxidation in 40 mL of 1 m KOH electrolyte at a scan rate of 5 mV s^−1^. Reproduced with permission.^[^ ^41^ ^]^ Copyright 2020, American Chemical Society.

Oxidation of amines produces aldehydes, ketones, nitriles, or other carbonyl compounds.^[^ ^104^ ^]^ During aromatic electrochemical cyanation, cationic active species are produced at the anode and attacked by cyanide anions to produce cyanation products. Generally, lowering the oxidation potential or increasing the carbocation density of cationic active species is a widely used approach, and choosing the appropriate aromatic ring substitute can determine the efficiency of cyanation.^[^ ^105^ ^]^ Nitrogen surfaces absorb amines and cause anodic oxidation, which results in various spatial orientations. The carbon atoms will be positioned closer to the electrode surface when the five‐membered amines are more planar, leading to the main products being *α*‐cyano. Additionally, cyanation on alkyl substituents in the following order would explain why tertiary amines with alkyl substituents other than methyl were the most important products: methyl>ethyl>propyl>isopropyl.^[^ ^106^ ^]^ Using NiO_x_ thin films as anodes, Xue et al. oxidized n‐butylamines to n‐butyronitriles in electrolytes containing 0.5 m K_2_SO_4_ and 0.5 m n‐butylammonium sulfate (BAS) at pH 12.^[^ ^107^ ^]^ Zhang et al. reported the successful oxidation of octylamine on a Ni_2_P NS array using 0.5 mol L^−1^ NaHCO_3_.^[^ ^108^ ^]^ Zhao and coworkers converted benzylamine into benzonitrile using 2D–conductive MoFs (2D cMOFs) in 1.0 m KOH.^[^ ^41^ ^]^ With a typical graphite electrode modified with TEMPO, it has been found that imine intermediates are oxidized to nitrile in anhydrous conditions and are hydrolyzed into carbonyl compounds in water during the amine electrooxidation reaction pathways.^[^ ^109^ ^]^ Cai and colleagues invented N‐cyanation of secondary amines and the cyanation of tertiary amines without transition metals.^[^ ^110^ ^]^ As a solvent, acetonitrile is used to prepare the corresponding cyanated products using the cyano source trimethylsilyl cyanide, additives KF, and tetrabutylammonium bromide. A study of the N‐cyanation process showed that tetrabutylammonium bromide played an important role, indicating that free radicals played a role in the process. Xiang et al. devised an oxidant‐free method using a bimetallic Ni/Co metal‐organic framework derivative (t‐Ni/Co MOF) as the anodic electrocatalyst for oxidizing benzylamine to benzonitrile (**Figure** **8**a).^[^ ^40^ ^]^ When utilizing substrates 1d and 1e, the oxidation rate of benzylamine to benzonitrile is lower for t‐Ni/Co MOF electrodes than that of monometallic Ni catalysts. Ni species develop quicker during reversible electron transfer between Co^2+^ and Co^3+^ at low potentials, speeding the kinetics of amine oxidation and lowering the energy consumption of hydrogen generation. The t‐Ni/Co MOF electrode can also be utilized to oxidize different primary amines with high yields (2b–2 g, 80–94%) in a membrane‐free cell, as demonstrated in Figure 8b. Wang et al. created a multimetallic 2D–conductive metal‐organic framework (2D cMOF) which exhibited high performance in electrochemical benzonitrile synthesis (Figure 8c).^[^ ^41^ ^]^

**Figure 9:**
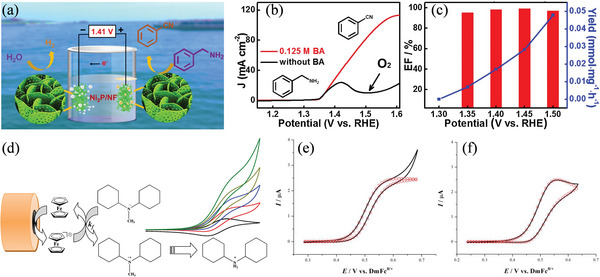
a) Electrocatalytic oxidation of benzonitrile over Ni_2_P/Ni nanomeshes. b,c) LSV curves yield rate and FE of BOR over Ni_2_P‐UNMs/NF nanocomposites. Reproduced with permission.^[^ ^112^ ^]^ Copyright 2019, Elsevier. d) Ferrocene‐mediated dealkylation of amines. Comparison of experimental (−) and simulated (○) CVs obtained with a scan rate of 0.02 V s^−1^ (1.0 mm diameter) for the oxidation of e) 0.75 mm Fc in acetonitrile (0.1 m [Bu_4_N][PF_6_]) in the presence of 20 mm DMCHA and f) 0.85 mm Fc in the presence of 2.9 mm DCHMA. Reproduced with permission.^[^ ^113^ ^]^ Copyright 2019, American Chemical Society.

Multimetallic 2D cMOFs exhibit superior electrooxidation performance of benzylamine (BA) due to their intrinsic electrically conductive 2D structure and the optimized multimetallic coupling catalytic effect. Wang et al. described a novel electrochemical approach for oxidizing BA on a series of multimetallic 2D MOF nanowires.^[^ ^41^ ^]^ The multimetallic 2D cMOFs, particularly the three metal 2D cMOFs (NiCoFe‐CAT, 2,3,6,7,10,11‐hexahydroxybenzophenanthrene), have intrinsic structural advantages that allow them to oxidize BA under alkaline conditions. Under moderate anode conditions, conversion of BA to BN is possible with high yields and FE. In 1.0 m KOH and 10 mm BA, an electrode current density of 10 mA cm^−2^ is reached with just a voltage of 1.29 V, which is better than most reported BA oxidation catalysts (Figure 8d). Huang et al. established a simple yet effective strategy for increasing hydrogen generation by substituting the OER with the oxidation of primary amine on a NiSe nanorod electrode in water, which significantly enhanced the selective conversion of primary amines to corresponding nitriles at the anode.^[^ ^111^ ^]^ As hydrophobic nitriles float easily on electrolytes, they can be synthesized on a continuous scale without catalyst deactivation. Ding et al. synthesized Ni_2_P‐UNMs/NF by phosphidating ultra‐thin Ni(OH)_2_ nanomeshes on NF substrates (Ni(OH)_2_‐UNMs/NF).^[^ ^112^ ^]^ The Ni_2_P‐UNMs/NF nanocomposites in an alkaline electrolyte show exceptional activity in both the HER and the BA oxidation reactions owing to the existence of enough active sites in the holes and their high specific surface area, porous framework, and NF substrate's high conductivity (**Figure** **9**a). In the presence of benzylamine, Ni_2_P‐UNMs/NF can be used directly as an electrochemical water‐splitting catalyst, requiring only a voltage of 1.41 V to generate a current density of 10 mA cm^−2^ in alkaline electrolytes and achieving high benzylamine yield rates and FEs at the anode (Figure 9b,c) In the experiment shown in Figure 9d, Torriero et al. investigated homogeneous catalytic oxidation of four compounds: dicyclohexylamines (DCHA), N,N‐dimethylcyclohexylamines (DMCHA), and N,N‐dicyclohexylmethylamines (DCHMA).^[^ ^113^ ^]^ The catalytic efficiency and the second‐order rate constants were found following the order DCHA ≪ DMCHA < DCHMA. Experiments obtained show excellent agreement with the simulations over a wide range of catalyst and amine concentrations. (Figure 9e,f).

### 2.4 Oxidative Coupling Reaction

**Figure 10:**
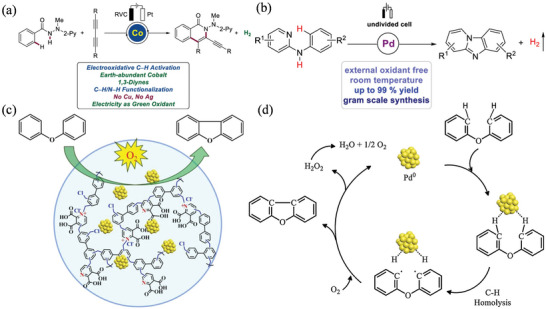
a) Cobalt electrocatalyzed oxidative C—H/N—H activation with 1,3‐diynes by electro‐removable hydrazides. Reproduced with permission.^[^ ^121^ ^]^ Copyright 2019, American Chemical Society. b) Pd‐catalyzed electrooxidative C—H amination. Reproduced with permission.^[^ ^122^ ^]^ Copyright 2020, American Chemical Society. c) Size and stability modulation of Pd NPs on porous hyper crosslinked ionic polymer for heterogeneous aerobic oxidative coupling of diaryl ether. d) Reaction route. Reproduced with permission.^[^ ^35^ ^]^ Copyright 2020, Elsevier.

It was previously possible to produce imines by condensing amines and carbonyl compounds along with Lewis acid catalysts.^[^ ^114^ ^]^ However, the homogeneous catalysts could not be re‐used in these circumstances.^[^ ^115^ ^]^ Dong et al. produced imines via bifunctional heterogeneous catalysis from BA, anilines, and benzylamines using Pd—Au@Mn^2+^‐MOFs.^[^ ^116^ ^]^ A variety of transition metal catalysts have been used for coupling reactions of amines, including low‐cost catalysts such as Cu, Mn, and Fe, and noble metals such as Pd, Au, and Pt.^[^ ^117^ ^]^ An Au—Pd catalyst supported on CNTs exhibited heterogeneous catalytic activity in aerobic amine oxidation with a 95% conversion ratio and 98% selectivity.^[^ ^118^ ^]^ As a clean, renewable energy resource, electrochemical synthesis of imines from amines is yet to be fully explored. Liu et al. showed that the oxidative coupling of amines can be used to synthesize imines and diazenes in an electronic‐promoted way without using metal catalysts or oxidants.^[^ ^119^ ^]^ To form C=N bonds and N=N bonds, the current and applied potential are adjusted. The method produces good yields of the desired compounds when conducted in air. Geng et al. obtained a Fe oxide catalyst by modulating the crystal phase and screening that was active and robust for oxidative coupling in imine synthesis.^[^ ^120^ ^]^ Unlike Fe_3_O_4_, atoms of *γ*‐Fe_2_O_3_ transfer electrons easily to form O_2_ and O^2−^ species when exposed to molecular oxygen, leading to good activity and selectivity. It is also possible to couple terminal alkynes and arylboronic acids using an Ag anode. A sequential reaction system produces several *π*‐extended butadiynes in one‐sequence by switching between oxidative and neutral conditions with electricity. Thienoacene is also synthesized electrochemically via intramolecular C—S coupling using Bu_4_NBr as the halogen mediator. Additionally, it is possible to synthesize gram‐scale products by electro‐oxidatively forming C—N bonds, and this method is employed to functionalize bioactive molecules in the late stages of their life cycle. Mei et al. achieved an efficient electron‐oxidative C—H/N—H activation using 1,3‐diynes and a robust cobalt catalyst (**Figure** **10**a).^[^ ^121^ ^]^

The electro oxidative C—H/N—H activation was performed in a simple undivided cell with remarkable functional group compatibility and ample scope for electrochemical C—H functionalization. Using this protocol, C—H activation is achieved without the use of stoichiometric and costly chemical oxidants with hydrogen as the only by‐product. Pyrido[1,2‐a]benzimidazoles were prepared using a simple, mild, and green process, which was the first reported catalyst for electrooxidative intramolecular C—H/N—H annulation without using external oxidants or additives (Figure 10b).^[^ ^122^ ^]^ It was found that Pd° could be recycled by oxidizing it on the anode. Wang et al. reported the high catalytic activity of heterogeneous Pd^0^ NPs in intramolecular oxidative couplings of diaryl ether with O_2_ (Figure 10c).^[^ ^35^ ^]^ When the ionic moiety content of Pd^0^ NPs is moderate, the constructed particles are highly effective in coupling diphenylether to dibenzofuran, resulting in a high yield of 72% and an increase in TOF of 91, which exceeds 18‐fold that observed with homogenous palladium acetate. Kinetic analysis and radical quenching results demonstrate the carbon radical catalytic pathway (Figure 10d). In a recent study, Chowdhury et al. demonstrated an electrochemical method for the direct cross‐dehydrogenation of benzylic C(sp^3^)—H bonds of toluidines with alcohols in the absence of oxidizing agents and bases.^[^ ^123^ ^]^ Nitrogen‐containing heterocyclic compounds and aniline derivatives have successfully been tested. The authors expect that this electrooxidative C(sp^3^)—H arylation will provide an attractive strategy for developing radical cross‐coupling chemistry in the future.

## 3 Advanced Electrocatalysts for Cathodic Reactions

Nanostructured materials have generated significant interest in cathodic electrocatalysis due to the micro‐hierarchical structure and surface characteristics (**Table** **2**). The catalyst surface shape has been recognized as one of the most crucial elements affecting catalyst activity and selectivity.^[^ ^124^ ^]^ To improve the electrochemical reduction activity, altering the crystal shape to expose the active catalytic sites better is usually required.^[^ ^125^ ^]^ This method offers energetically favorable sites for the adsorption of the intermediate of reduction needed. For instance, the catalysts offering a higher ratio of active stepping sites on the higher preferred facets, were credited with the increased activity.^[^ ^126^ ^]^ However, due to the increased surface energy compared to the bulk material during electrocatalytic reduction, nanosized particles rarely succeed in maintaining their structure.^[^ ^127^ ^]^ More studies have linked the observed decline in catalyst activity and selectivity to modifications of the existing surface that change the structure of the active sites, despite some studies mentioning the importance of surface reconstruction for providing a fresh available catalyst surface and improving its electrochemical properties.^[^ ^128^ ^]^

**Table 2:** Comparison of electrocatalytic reduction performance of various catalysts

Catalyst	Electrolyte	Substrate	Product	*η*_org._	FE [%, Org.]	Ref.
Cu/TNT^a)^	0.5 m NaHCO_3_	CO_2_	MeOH	−2.0 V vs RHE	5 for MeOH	[128a]
Cu_2_O_(OL‐MH)_/Ppy^b)^	0.5 m KHCO_3_	CO_2_	MeOH	−0.85 V vs RHE	93 ± 1.2 for MeOH	[125a]
Cu_2_O on Cu foils	0.5 m KHCO_3_	CO_2_	MeOH	−1.1 V vs SCE	38	[125b]
Cu_2_O/ZnO	0.5 m KHCO_3_	CO_2_	MeOH, EtOH	1.85 V vs RHE	27.5 for MeOH, and 3.9 for EtOH	[129]
Cu_2_O‐o^c)^	0.5 m KHCO_3_	CO_2_	MeOH, EtOH, 2‐propanol	−0.3 V vs RHE	4.9 for MeOH, 17.9 for EtOH, and 12.6 for 2‐propanol	[125c]
CuO/TiO_2_	0.5 m KHCO_3_	CO_2_	EtOH, n‐propanol	−0.85 V vs RHE	36.8 for EtOH, and 5.8 for n‐propanol	[130]
P‐doped Cu (P 8.3%) (Cu_0.92_P_0.08_)	1 m KOH	CO_2_	EtOH	−0.7 to −0.75	64 C_2+_ (15 EtOH)	[128b]
Ag@Cu core‐shell	0.1 m KHCO_3_	CO_2_	CO, CH_4_, C_2_H_4_	−1.06 (vs. RHE)	82.00	[125d]
Oxide‐derived‐Pb films	0.5 m NaHCO_3_	CO_2_	Formate	−0.75 V vs RHE	ca. 100	[128c]
Sn‐doped Ga_2_O_3_ (Gallium oxide) films	3 m KCl and 5 m NaOH	CO_2_	Formate	−1.6 V vs Ag/AgCl	80	[128d]
Cu nanoflower electrode	0.1 m KHCO_3_	CO_2_	Formate	−1.2 V vs RHE	40	[125e]
Cu nanofoams	0.1 m KHCO_3_	CO_2_	Formate	−1.5 V vs Ag/AgCl	37	[125f]
Sn/SnO_x_ thin‐film	0.5 m NaHCO_3_	CO_2_	Formate	−0.7 V vs RHE	19	[131]
AuPd alloys film	0.1 m KHCO_3_	CO_2_	Formate	−0.88 V vs RHE	10	[127b]
BDD electrode^d)^	MeOH	CO_2_	Formaldehyde	−1.7 V vs Ag/AgCl	74	[128e]
Cu NPs electrode	0.1 m KClO_4_	CO_2_	C_2_H_4_	−1.1 V vs RHE	36	[127c]
Cu_2_O‐derived copper NPs	0.1 m KHCO_3_	CO_2_	C_2_H_4_	−1.1 V vs RHE	ca. 33	[128f]
Fe‐Ni/rGO/Ni foam^e)^	0.05 m DMF_sat_ with 0.1 m Na_2_SO_4_	Trichloro‐ethylene	C_2_H_4_		78.8	[127d]
N‐doped nanodiamond on Si rod array	0.5 m NaHCO_3_	CO_2_	Acetate	−0.8 V vs RHE	77.3	[126b]
polyaniline/Cu_2_O nanocomposite‐based electrode	0.1 m TBAP in MeOH	CO_2_	Acetate	−0.3 V vs SCE	63	[128g]
Defect‐Site‐Rich Cu Surface	0.1 m KClO_4_	CO_2_	C_2+_ alcohols	−3.5 V	≈70	[132]
Dendritic Cu	1 m KOH	CO_2_	C_2+_, C_2_H_4_, EtOH		85.2 C_2+_ (35.5 C_2_H_4_, 38.0 EtOH)	[126a]
Reconstructed porous Cu	0.1 m KHCO_3_	CO_2_	C_2_	−1.09	80 C_2_	[124f]
CuO_x_	0.1 m CsHCO_3_	CO_2_	C_2+_	−0.9	ca. 80 C_2+_	[124e]
Cu‐oxide‐/hydroxide‐derived	0.1 m KHCO_3_	CO_2_	C_2+_	−1.05	ca. 70 C_2+_	[127e]
Cu‐NPs + polyaniline	0.1 m KHCO_3_	CO_2_	C_2+_ with C_2_H_4_, (EtOH, PrOH)	−1.2	80 C_2+_ with 40 C_2_H_4_, (EtOH, PrOH)	[124d]
Multihollow Cu oxide	2 m KOH	CO_2_	C_2+_	−0.61	75.2 C_2+_	[124c]
[Ni(bpy)_3_]^2+^ ^f)^	DMF 0.1 m nBu_4_NPF_6_	Alkynes (3‐phenylprop‐2‐yn‐1‐ol)	Alkenes	−1.9 V_FC_	97.9	[124b]
Pd@ArS‐Pd_4_S NTs^g)^	1 m KOH	4‐ethynylaniline	4‐vinylaniline	−1.1 V vs Hg/HgO	75	[127f]
Cu—S NSs^h)^	1 m KOH	4‐ethynylaniline	4‐vinylaniline	−1.3 to −1.4 V vs Hg/HgO	99	[124a]

^a)^ copper doped titanium oxides nanotubes

^b)^ The Cu_2_O/Ppy particles possessing both octahedral and microflower shapes with exposed low‐index (111) facets and high‐index (311) and (211) facets are denoted as Cu_2_O_(OL‐MH)_/Ppy particles

^c)^ octahedron structure of Cu_2_O with (111) facets

^d)^ boron‐doped diamond

^e)^ Fe‐Ni/reduced graphene oxide/Ni foam

^f)^ Tris(2,2′‐bipyridyl)dichlororuthenium(II) hexahydrate

^g)^ Pd@carbon‐supported sulfur anions and thiolate‐modified Pd nanotips

^h)^ surface sulfur‐doped and ‐adsorbed Cu nanowire sponges.

### 3.1 Reduction of Carboxylates

The electro‐carboxylation process for CO_2_ immobilization is both environmentally friendly and technologically feasible. Olefins, alkynes, alcohols, aldehydes, ketones, epoxides, imines, and organic halides can be carboxylated using electrocatalysis.^[^ ^133^ ^]^ In electrochemical carboxylation, substrates were reduced to CO_2,_ or carboxylate.^[^ ^134^ ^]^ Sacrificial cathodes are frequently used for such reactions, as they provide counterion while preventing the unwanted oxidation of products. A method of making succinic acid derivatives by dicarboxylation of styrenes has been described by Senboku and colleagues.^[^ ^135^ ^]^ High to exceptionally high yields of dicarboxylated products have been obtained using Pt cathode and Mg sacrificial anodes. The reduction potentials of CO_2_ and styrenes are precisely the same, based on CV experiments, which could suggest two possible reactions. Styrenes with electron neutral or donating groups reduce CO_2_ preferentially (2.53 V vs Ag/Ag^+^), resulting in an anion of CO_2_ that can add to olefins.^[^ ^136^ ^]^ By contrast, olefin reduction dominates in styrenes with a lower absolute reduction potential (compared to CO_2_), and CO_2_ can capture the resultant carbanion. The two processes are capable of running simultaneously. In follow‐up research, electrode types, electrolytes, temperatures, and other variables were also examined.^[^ ^137^ ^]^ Jiao et al. reported an electrocarboxylation mechanism that uses palladium catalysts to produce *α*‐aryl carboxylic acids selectively and efficiently.^[^ ^138^ ^]^ Cinnamyl acetate undergoes asymmetric carboxylation, despite moderate enantioselectivity. A mesoporous Ag material was used as an electrocatalyst to perform halogenation reactions.^[^ ^139^ ^]^ The mesoporous Ag is composed of mesoporous particles of uniform size (8 nm), which obtains a high performance (78%) towards electrocatalytic carboxylation of halogenated compounds to acid.

**Figure 11:**
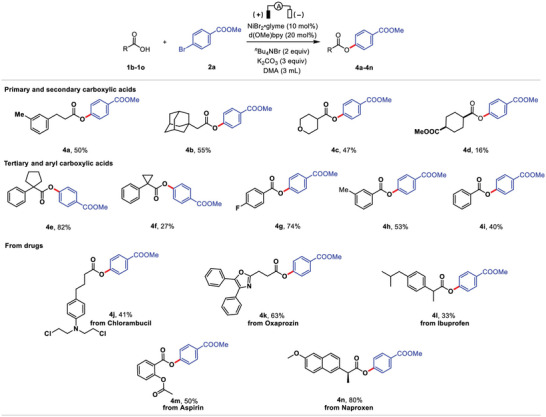
Evaluation of carboxylic acids (The preparative utility of this electrochemical esterification reaction was demonstrated by using a reaction containing 5 mmol of substrate 2a, which furnished desired product 3a in 48% yield). Reproduced with permission.^[^ ^140^ ^]^ Copyright 2021, American Chemical Society.

Wei et al. performed the esterification of carboxylic acid with aryl halide in an undivided electrochemical cell using a Ni catalyst.^[^ ^140^ ^]^ The reaction was well tolerated by various functional groups, as shown in **Figure** **11**. A wide range of options among the pri‐, sec‐, tert‐, and aryl acids (4a–4i) can be used for this synthesis. Several drug molecules are subjected to the optimized conditions to demonstrate this protocol's utility. This transformation is also carried out by chlorambucil containing alkyl chloride (4j). Oxaprozin (4k) is a good yielding product when combined with nitrogen‐ and oxygen‐containing heterocycles. Ibuprofen and naproxen (4l and 4n), two carboxylic acids with activated positions, are all converted into esterified analogs.

**Figure 12:**
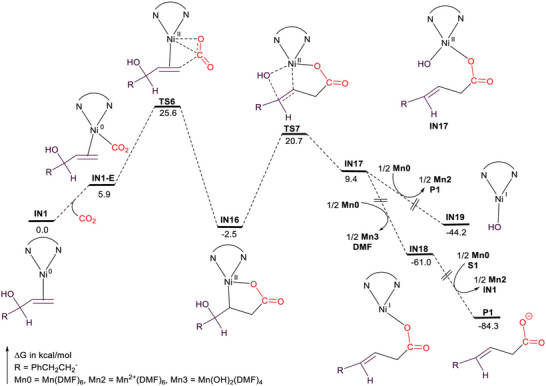
Ni‐catalyzed regioselective carboxylation of allylic alcohols. Gibbs free energy profiles of the carboxylation—C—O activation pathway. Reproduced with permission.^[^ ^145^ ^]^ Copyright 2021, American Chemical Society.

Carbin et al. utilized a salt containing an inorganic cation that prevents nucleophilic processes from preserving selectivity without a sacrificial anode.^[^ ^141^ ^]^ Anhydrous magnesium bromide is used as a cheap, soluble source of magnesium cations to achieve moderate to good yields (34–78%) in the carboxylation of a wide range of aliphatic, benzylic, and aromatic halides. The yields from sacrificial‐anode‐free methods are generally equivalent to or better than yields from standard sacrificial‐anode techniques. Analyzing a wide range of substrates indicates a link between carbon–halide bonds' nucleophilic susceptibility and reduced selectivity when no Mg^2+^ source is available. These findings constitute a critical step toward developing sustainable and practical carboxylation by offering an electrolyte design guideline that eliminates the requirement for sacrificial anodes. Direct acylation of benzylic C(sp^3^)—H bonds in alkyl carboxylic acids can be used to produce dialkyl ketones from these commercially available alkyl carboxylic acids in good yields. Using Pd, an electro‐carboxylation method has been developed for regioselectively forming *α*‐aryl acids from homostyrenyl acetates.^[^ ^138^ ^]^ Dharmaratne et al. used pyrolytic graphite‐edge electrodes modified with multiwalled carbon nanotubes‐1‐pyrenebutyric acid (MWNTPy), MWNTP‐COOH/Py, or MWNTP‐COOH alone to activate carbodiimides and inhibit bilirubin oxidase (BOD) formation.^[^ ^142^ ^]^ While the MWNT—COOH/Py electrode shows the highest relative amount of surface —COOH groups, it also shows the largest oxygen reduction current when immobilized with BOD compared to others. One of the underlying factors for the observed electrocatalytic trend is that hydrophobic MWNT surfaces interact better with electron‐received T1 Cu sites than more polar and less defective MWNT—COOH materials. Li et al. developed a method of synthesizing monofluoromethyl vinyl compounds by decarboxylating monofluoromethyl vinyl with CF_3_SO_2_Na.^[^ ^143^ ^]^ This electrochemical decarboxylative trifluoromethylation is extremely stereoselective and produces high‐quality products with a variety of substrate compatibility in non‐metallic and external chemical oxidant‐free conditions. Yang et al. fabricated electrospinning nitrogen‐doped carbon nanofibers embedded with platinum NPs onto flexible CC composites, which were used as highly efficient and binder‐free, and stable catalysts for CO_2_ reduction.^[^ ^144^ ^]^ As a result, 2‐phenylpropionic acid with 99% yield and formate with 91% efficiency is obtained. Liu et al. calculated the process of carboxylation of allylic alcohols by Ni‐catalyzed reactions in detail with DFT (**Figure** **12**).^[^ ^145^ ^]^ The activation of allylic alcohol, oxidative ligation, reduction, and carboxylation processes are discovered to be involved in the reaction. The rate‐determining step is the first, and moisture in the reaction system plays a critical role. Proton‐relay generates allylic hydrogen carbonate and undergoes further oxidative ligation processes by forming hydrogen bonds between water and substrate. However, regioselectivity on the terminal carbon atom is mostly determined by steric hindrance between CO_2_ and the allylic group, whereas E/Z selectivity is predominantly determined by the E‐substrates' thermodynamic stability. Carboxylation—C—O is activated, resulting in IN1‐E, which then proceeds carboxylation with an energy barrier of 19.7 kcal mol^−1^. The next C—O bond cleavage of the Ni^2+^ metallacycle intermediate IN16 occurs through the transition state TS7, with an energy barrier of 23.2 kcal mol^−1^. The produced intermediate IN17 can be dehydroxylated to yield the Ni^+^ species IN18 in the presence of the Mn reductant (IN19 could be eliminated due to its relatively higher energy). P1 and IN1 are released and regenerated once IN18 is reduced and S1 is coordinated. Because the transition state TS6 had a larger energy than the initial C—O activation pathway, a carboxylation‐C—O activation pathway was ruled out.

CO_2_ and the diene are likely both reduced catalytically, regardless of the exact mechanism. In the electro‐reduction of CO_2_ reaction (eCO_2_RR), one electron may be achieved electrochemically by carboxylation of butadiene with a Ni‐based mediator.^[^ ^146^ ^]^ Duach described the carboxylation of alkynes to produce propionic acid derivatives using Ag cathodes.^[^ ^147^ ^]^ Terminal alkynes are deprotonated with an electrogenerated base and terminal acetylides are trapped with CO_2_. Internal alkynes are not carboxylated under the conditions of the reaction. Ag cathode and electrogenerated acetylides formed a strong contact, which improved the selectivity. Jiang et al. demonstrated that terminal alkynes can be dicarboxylated under various reaction conditions, resulting in the formation of maleic anhydride derivatives.^[^ ^133e^ ^]^ By increasing CO_2_ pressure and injecting catalytic amounts of CuI, a product called tricarboxylate is formed.^[^ ^148^ ^]^ The electro‐reduction process is essential for generating an alcoholic molecule from a carboxylic acid and storing direct electricity in a water‐soluble chemical, that is, a carrier liquid that is easy to transport. Recently, using anatase TiO_2_ electrode, Sadakiyo et al. synthesized glycolic acid, a monovalent alcoholic molecule.^[^ ^149^ ^]^ It is still rare to find carboxylic acid alcohols from oxalic acid that do not generate aromatic carboxylic acids. Ketones and CO_2_ have similar reduction potentials, which are thought to reduce in galvanostatic conditions. Through reductive carboxylation of ketones, it is possible to make *α*‐hydroxycarboxylic acids.

### 3.2 Reduction of CO_2_ and CO

eCO_2_RR is crucial to synthesize CO, methanol, hydrocarbons, and formate, which provides a safer and more environmentally friendly means of storing intermittent renewable energy and helping to reduce CO_2_ emissions.^[^ ^150^ ^]^ Metal catalysts for eCO_2_RR play an essential role in industrial development.

#### 3.2.1 Reduction of CO_2_ to Alcohols

Wang et al. created pyridine‐derived organically doped bimetallic PdCu catalysts to achieve the transformation of CO_2_ into alcohol.^[^ ^151^ ^]^ 4‐[3,2‐bis(phenoxymethyl)‐2,2‐propoxy]pyridine (PYD) is entrapped within a PdCu alloy to form PYD@PdCu. The catalyst achieves 26% and 12% FEs that utilize 0.04 and 0.64 V to generate methanol and ethanol, respectively. It has been found that the Cu component aids ethanol production, while PYD aids methanol synthesis. Chen et al. found that a composite made of nitrogen‐doped graphene quantum dots on CuO‐derived Cu nanorods (NGQ/Cu‐nr) was extremely effective for reducing CO_2_ to ethanol and n‐propanol.^[^ ^152^ ^]^ A total current density of 282.1 mA cm^−2^ and 52.4% FE of C_2+_ alcohols are achieved, which highlights the superiority of the catalyst. The NGQ/Cu‐nr significantly accelerated the eCO_2_RR to alcohols by offering dual catalytic active sites and promoting further carbon protonation as a result of the synergistic effects between NGQ and Cu‐nr. At various catalytic loadings and weight ratios, the generation of CH_3_OH from CO_2_ liquid phase electroreduction is assessed at Cu_2_O‐ZnO combinations.^[^ ^129^ ^]^ To get beyond the mass transfer limitation, GDEs with spray‐supported Cu_2_O and Cu_2_O/ZnO catalysts are used for the continuous electroreduction of CO_2_ in the gas phase, resulting in great CO_2_RR performance.^[^ ^130^ ^]^ CuO/TiO_2_ with an anticipated CuO concentration of 60% was also used as an effective electrocatalyst for CO_2_RR in 0.5 m KHCO_3_ solution, demonstrating the most unusual activity (total FE of 47.4% at a potential of 0.85 V vs RHE). The Cu catalyst was synthesized rationally by Gu et al. in a CO‐rich environment to encourage the formation of defect‐rich sites that are ideal for CO adsorption.^[^ ^132^ ^]^ These defect‐rich sites provide a high surface density of adsorbed CO intermediates during the electrochemical CO_2_ reduction process, optimizing the CO_2_ electroreduction pathways toward synthesizing C_2+_ alcohols.

**Figure 13:**
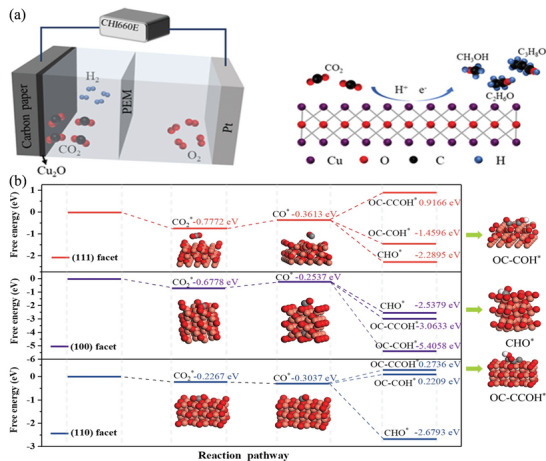
a) eCO_2_RR over Cu_2_O nanostructures. b) Free energy in eCO_2_RR on (111), (100), and (110) crystal facets of Cu_2_O catalysts. Reproduced with permission.^[^ ^125c^ ^]^ Copyright 2021, American Chemical Society.

Lv et al. developed a Cu_3_Ag_1_ electrocatalyst by replacing the electrodeposited Cu matrix with a galvanic film.^[^ ^153^ ^]^ As a result of the interphase electron transfer from Cu to Ag, electron‐deficient Cu sites are generated. Benefiting from the electron‐deficient property, the Cu_3_Ag_1_ electrocatalyst achieves a FE of 63% and an alcohol partial current density of 25 mA cm^−2^ at 0.95 V compared to the copper‐bare electrode matrix, corresponding to a 126‐fold improvement in selectivity and a 25‐fold increase in activity. Nanosized Cu_2_O catalysts with a variety of morphologies and crystal phases such as Cu_2_O‐c (cubic structure with 100 facets), Cu_2_O‐o (octahedron structure with 111 facets), Cu_2_O‐t (truncated octahedron structure with (100) and (111) facets are synthesized by controlling the density of a polyvinyl pyrrolidone (PVP) template for eCO_2_RR to alcohols (**Figure** **13**a).^[^ ^125c^ ^]^ Using Cu_2_O synthesized, methanol, ethanol, and 2‐propanol with a FE of 4.9%, 17.9%, and 12.6%, respectively, can be produced by reducing CO_2_. The alcohol selectivity of the different Cu_2_O NPs follows the order: Cu_2_O‐t > Cu_2_O‐u > Cu_2_O‐c > Cu_2_O‐o. Facet‐dependent effects are observed due to differences in oxygen‐vacancy defects and CO_2_ reduction energy barriers. Figure 13b displays the CO^*^ intermediate's free energies on (111), (100), and (110) facets of Cu_2_O. CO^*^ is more strongly adsorbated on the (111) facet than on the (100) or the (110) facets. Thus, the facet (111) promotes the adsorption and stabilization of CO^*^ intermediates, increasing C—C coupling during the ECR process. Moreover, it has negative free energy on the (111) facet for CHO^*^ and C_2_O_2_H^*^ intermediates, while the C_3_O_2_H^*^ intermediate presents positive free energy, which suggests that an extra amount of energy is necessary to trigger the protonation of CO^*^‐coupled electron transfer.

**Figure 14:**
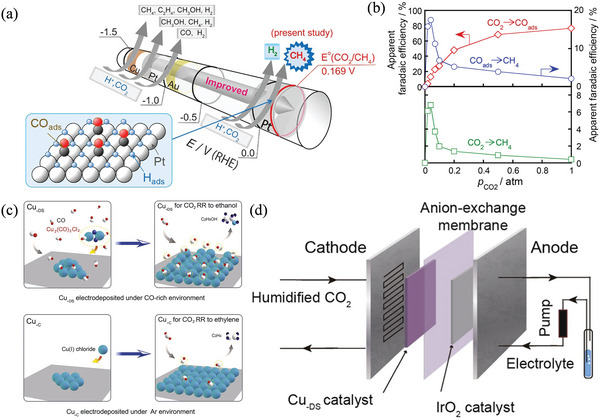
a) eCO_2_RR to methane on platinum catalysts without overpotentials. b) Upper half; Apparent FE of the initial eCO_2_RR to adsorbed CO (red) and of the subsequent reduction of adsorbed CO to methane (blue). Lower half; Apparent FE of the overall reaction (CO_2_ to methane, green) calculated by using the apparent FSs of the two successive processes. Reproduced with permission.^[^ ^156^ ^]^ Copyright 2019, American Chemical Society. c) Schematic of efficient eCO_2_RR to C_2+_ alcohols at defect‐site‐rich Cu surface. d) Schematic diagram of an MEA electrolyzer. Reproduced with permission.^[^ ^132^ ^]^ Copyright 2020, Elsevier.

Sun and colleagues developed multiple Pd_x_Cu_y_ aerogels by reducing metal precursors in situ and using supercritical CO_2_.^[^ ^154^ ^]^ Pd_83_Cu_17_ airgel shows an 80% MeOH generation rate, with an electrocatalytic efficiency of up to 83% and a current density of 31.8 mA cm^−2^. The excellent efficiency and selectivity are ascribed to the effective adsorption and stabilization of the CO_2_ radical anion and the high Pd^0^/Pd^2+^ and CuI + Cu^0^/Cu^2+^ ratio of the aerogel structure. Wolff et al. reviewed molecular electrocatalytic systems for the hydrogenation and dehydrogenation of carbonyls and alcohols.^[^ ^155^ ^]^ They emphasize the importance of key mechanistic concepts for linking with more mature schemes for transfer hydrogenation, proton reduction, and CO_2_ reduction. Umeda et al. reported a selective reduction of adsorbed CO to methane using carbon‐supported Pt catalysts and, more importantly, at potentials close to thermodynamic equilibrium (**Figure** **14**a).^[^ ^156^ ^]^ Although the apparent FE is not high enough for commercial applications, it is the first demonstration of electrochemical methane generation without an overpotential. Figure 14b (top) shows the FE in relation to the CO_2_ level. In addition to increasing faradaic CO_ad_ formation to 80% with increasing pCO_2_, the apparent Faradaic methane reduction efficiency of CO_ads_ increased to 17.5% at pCO_2_ = 0.04 atm before decreasing sharply at higher pressures due to the self‐poisoning effect of CO_ads_. Taking the experimental data in Figure 14b (top) and calculating the apparent FE with QCH_4_/(QCO + QCH+ QH + Qdl) × 100% (Figure 14b, bottom), the apparent FE of this reaction is 6.8% at pCO_2_ = 0.04 atm. Gu et al. achieved ≈70% FE for C_2+_ alcohols (Figure 14c).^[^ ^132^ ^]^ Specifically, they use a CO‐rich environment to fabricate Cu catalysts with stepped sites that enable high surface coverage of ^*^CO intermediates as well as bridge‐bound ^*^CO adsorption, allowing CO_2_RR pathways to be triggered and alcohols to be formed. The C_2+_ alcohols are produced in the flow‐cell electrolyzer and the membrane electrode assembly (MEA) electrolyzer are both enhanced by this defect‐site‐rich Cu catalyst. Cu—C remains highly selective to C_2_H_4_ in flow cells, however. As compared to Cu—C, Cu—DS increases the alcohol‐to‐ethylene conversion efficiency by 54‐fold. An electrolyzer equipped with Cu—DS is powered by 3.5 volts at a full‐cell voltage of 5 cm^2^ for a continuous 30‐h test (Figure 14d) to demonstrate stable CO_2_‐to‐alcohol conversion in a continuous manner.

**Figure 15:**
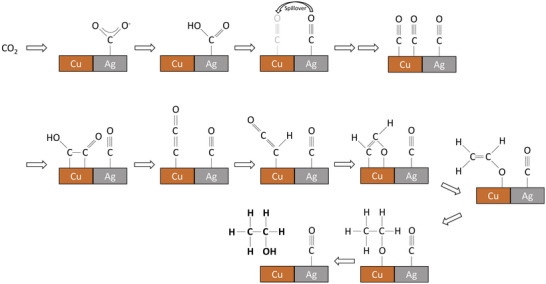
Proposed mechanism for eCO_2_RR to CO, followed by ethanol formation at bimetallic Cu—Ag foam. Reproduced with permission.^[^ ^160^ ^]^ Copyright 1969, Elsevier.

Li et al. prepared Cu—Co—Zn—Al catalysts for direct hydrogenation of CO_2_ using coprecipitation synchronous aging.^[^ ^157^ ^]^ Based on the results of the catalytic performance evaluation, the liquid product gains 116 CO_2_ moles per kg catalysis h^−1^, with methanol, ethanol, propanol, and diisopropyl ether yields of 31, 41, 12, and 32 mol g^−1^ catalysis h^−1^, respectively. Mechanism study shows that Cu is crucial to activating the C=O bond of CO_2_ to form intermediates. Co^+^ and Co^1−^ play a critical role in C—C coupling, ZnO improves the dispersion and stability of Cu, and Al_2_O_3_ as support gains enhanced methanol synthesis and alcohol dehydration. These three factors are responsible for excellent performance. Zhao et al. investigated the catalytic mechanism of the eCO_2_RR on the IrO_2_ (110) electrocatalyst model using DFT calculations.^[^ ^158^ ^]^ Based on these findings, the CO^*^ spectator may be highly effective in promoting methanol production and having low sensitivity to CO_2_ reduction to methane. With a CO^*^ coverage of 50%, methanol and methane onset potentials are respectively −0.32 versus RHE and −0.68 versus RHE, which indicates that the selectivity of CO_2_ catalytic reduction to methanol can greatly improve with an appropriate CO^*^ coverage. A vital intermediary, CH_3_O^*^+OH^*^, is a branch point that leads to the formation of two products, methanol, and methane, in the major pathway to methanol and methane. Despite the fact that both protonation reactions are exothermic, methanol production is a more favorable option than CH_4_ formation. The results show that iridium dioxide should act well as a catalyst for eCO_2_RR, and CO^*^ spectators also exhibit an enhanced effect on the selectivity of CO_2_ reduced to alcohol products. Raaijman et al. investigate CO reduction in Ag in order to establish its ability to produce ethanol based on DFT.^[^ ^159^ ^]^ The discrepancy between DFT and experimental results (ethanol vs no ethanol) is currently being addressed by investigating CO reduction at higher surface coverage (by increasing pressure) to determine if desorption effects can account for the discrepancy. According to these results, ethanol and propanol are the main C_2+_ products, which means that Ag electrochemistry is similar to Cu by virtue of the acetaldehyde‐like intermediates. **Figure** **15** shows the proposed mechanisms for bimetallic catalysts. As a result of this process, CO_2_ is reduced to CO in the presence of Zn, Ag, Pd, or pyridinic N sites, where CO is weakly adsorbent and can migrate to copper sites. The CO is bound superiorly where is either reacted with or reduced further by adjacent intermediates ^*^C1 and ^*^C_2_. Due to an altered electronic structure, the ratio of Cu to Ag was expected to directly influence the product distribution in catalysts containing Cu and Ag. With Cu and Ag interacting, the Ed values are shifted from Cu toward Ag, which indicates a shift in the center of the d‐band. As a result of the electronic interaction between Cu and Ag, the phase‐blended Ag—Cu catalysts demonstrated a 34.2% selectivity for ethanol, which is three times higher than pure Cu_2_O.

#### 3.2.2 Reduction of CO_2_ to Hydrocarbons

**Figure 16:**
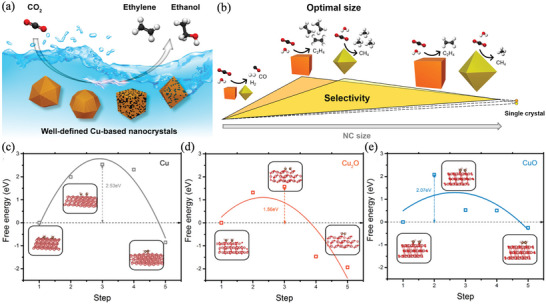
a) Well‐defined copper‐based nanocatalysts for selective eCO_2_RR to C_2_ products. b) Schematic illustration of size‐ and shape‐dependent behavior of Cu NCs in eCO_2_RR. Reproduced with permission.^[^ ^166^ ^]^ Copyright 2022, American Chemical Society. Reaction free energy diagram toward C_2_H_4_ from a pair of *CH_2_ on the surfaces of c) Cu, d) Cu_2_O, and e) CuO. Reproduced with permission.^[^ ^167^ ^]^ Copyright 2020, American Chemical Society.

Cu is a very effective catalyst for eCO_2_RR to hydrocarbons.^[^ ^161^ ^]^ Gong and coworkers observed that rhombic dodecahedral PdCu_3_ enhanced methane formation as a consequence of eCO_2_RR studies.^[^ ^162^ ^]^ This catalyst exhibits a 200 mV lower onset potential for methane than Cu foil and a sevenfold greater current density at 1.2 V, which is due to the high‐index facets and alloying. Using intramolecular cuprophilic interactions, Zhang et al. produced Cu^1−^‐based coordination polymers (NNU‐32) and integrated them into a CO_2_ flow cell electrolyzer, which displayed excellent selectivity for electrocatalytic CO_2_‐to‐CH_4_ conversion.^[^ ^163^ ^]^ The study looks at the impact of intrinsic coprophilic interactions in Cu^1−^‐based catalysts on eCO_2_RR electrocatalytic efficiency and provides a useful case study for developing more stable and efficient crystalline catalysts. A highly efficient heterostructured catalyst was developed by Lin et al., composed of a carbon nitride‐encapsulated copper oxide hybrid (Cu_x_O/CN).^[^ ^164^ ^]^ In such a heterostructure, the metal and CN interaction enhance the intrinsic electrical conductivity and the charge transfer processes at the metal–support interfaces. Despite the high C_2_H_4_ FE of 42.2%, these modified Cu‐based electrocatalysts offer remarkable enhancements in hydrocarbon selectivity and can also suppress the generation of H_2_ during the eCO_2_RR. The model of molecular catalysts for CO_2_ reduction proposed by Bao et al. utilized symmetric and asymmetric co‐ion porphyrins (PorCos), which all demonstrated promising eCO_2_RR properties.^[^ ^165^ ^]^ Increasing the amount of 2,6‐dimethylbenzene results in narrower band gaps for the complexes due to its electron‐donating effects. The asymmetric PorCo has the lowest onset potential of 288 mV and the highest FE of 93% at −0.6 V versus RHE. It ranks highest among all the reported state‐of‐the‐art porphyrin‐based electrocatalysts. By controlling the atomically positioned dimethylbenzene in PorCo, the CO_2_ reduction performance is significantly improved by stacking between PorCo with CNTs and adjacent PorCos. DFT calculations suggest that the electrical charge density between PorCo and CNT is highest due to the weak steric hindrance in as‐PorCo. Zaza et al., found that Cu‐based bimetallic NC (Cu NCs) could be optimized for the optimal face/edge interface size to maximize the selectivity toward certain products (**Figure** **16**a,b).^[^ ^166^ ^]^ Undersized segments at corners, steps, and kinks increase the undercoordinated sites' density, eventually promoting two electron‐reducing pathways, such as HER.

**Figure 17:**
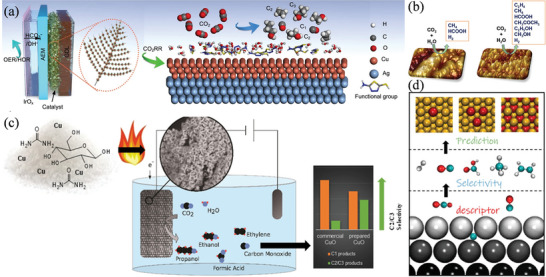
a) Illustration of the functionalized Ag—Cu electrodes in a membrane electrode assembly. Reproduced with permission.^[^ ^170^ ^]^ Copyright 2021, The Author(s). b) Product selectivity in CO_2_ electroreduction varies with Cu/Cu_2_O nanostructures and the direction of growth. Reproduced with permission.^[^ ^171^ ^]^ Copyright 2021, Elsevier. c) Selectivity of C_2_ and C_3_ products in eCO_2_RR on carbon‐doped copper oxide catalysts. Reproduced with permission.^[^ ^172^ ^]^ d) Using descriptors to predict the selectivity of different catalysts based on CO_2_RR. Reproduced with permission.^[^ ^173^ ^]^ Copyright 2021, American Chemical Society.

Wang et al. offered an approach for stabilizing Cu^*δ*+^ species by growing ZnO_x_ NPs on Cu foil to establish a Cu/ZnO_x_ interface to improve the eCO_2_RR selectivity.^[^ ^167^ ^]^ The interface stabilizes the surface Cu^2+^ species and provides remarkable methane selectivity (36%) and long‐term durability at a potential of −1.1 V (vs RHE). Based on the simulation trials and DFT calculations, they identified Cu^2+^ species as active locations for CH_4_ production while inhibiting the creation of ethylene. The challenge to producing multicarbon (C_2+_) liquid fuels using eCO_2_RR mainly is the difficulty of stabilizing reaction intermediates and controlling their subsequent C—C couplings. Using amorphous CuTi (a‐CuTi@Cu) as an eCO_2_RR catalyst, Hu et al. report eCO_2_RR to multicarbon (C_2–4_) liquid fuels.^[^ ^168^ ^]^ With a FE of 49% at 0.8 V versus RHE, the electrocatalyst makes ethanol, acetone, and n‐butanol the major products. Based on theoretical simulations and in situ testing, it has been shown that subsurface Ti atoms can enhance the electron density at surface Cu sites, resulting in enhanced adsorption of ^*^CO intermediates for dimerization and trimerization of ^*^CO. Gao et al. found that the heterogeneous structure between Cu_2_O and carbon should inhibit C_2_H_5_OH formation and facilitate C—C coupling, leading to the formation of C_2_H_4_.^[^ ^169^ ^]^ Wu et al. reported the improved conversion of CO_2_ into hydrocarbon by modifying the surface of bimetallic Ag—Cu catalysts using aromatic heterocycle derivatives such as thiadiazole and triazole derivatives (**Figure** **17**a).^[^ ^170^ ^]^ It has been discovered that the electron‐withdrawing properties of functional groups orient the reaction pathway toward the production of C_2+_ species (ethanol and ethylene) by adjusting the electronic states of Cu. Castro‐Castillo et al. exploited the effect of differential orientations of Cu facets on the eCO_2_RR product selectivity (Figure 17b).^[^ ^171^ ^]^ A Cu nanostructure with predominant (111) orientation yields 66.57% FE for methane at an applied potential of −1.3 V (vs RHE). Iwanow et al. showed how they used a thermal oxidation process of Cu‐containing deep eutectic solvent (DES) to make C‐doped CuO_2_ catalysts in Figure 17c.^[^ ^172^ ^]^ DES galactose‐urea catalysts constructed with Cu NPs and calcined for 60 min in air demonstrated increased selectivity towards C_2_ and C_3_ products. Liu et al. proposed the C monomer mobility and the CO_2_/CO adsorption energy are two effective descriptors (Figure 17d).^[^ ^173^ ^]^ Using the two descriptors, they further analyze a variety of alloy catalysts and identify which one may be most effective at eliminating CO_2_ from the atmosphere.

**Figure 18:**
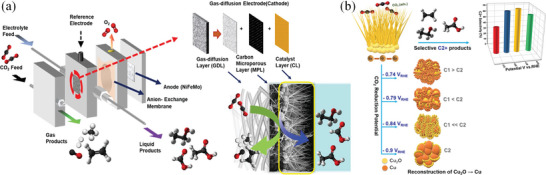
a) Illustration of GDEs for the eCO_2_RR and b) A possible mechanism for the formation of the selective C2+ products in the electrochemical CO_2_ RR using the etched Cu—Al electrode. Reproduced with permission.^[^ ^182^ ^]^ Copyright 2022, Elsevier.

eCO_2_RR to methane with the bimetallic catalyst was twofold greater in organic and aqueous solutions. The stability of intermediates can be tuned by controlling morphology,^[^ ^174^ ^]^ grain boundaries,^[^ ^175^ ^]^ facets,^[^ ^176^ ^]^ oxidation state,^[^ ^177^ ^]^ and dopants^[^ ^178^ ^]^ for reactions on copper by controlling grain boundaries, grain boundaries, faces, and oxidation states. A new design guideline has been provided by Zhang et al. for the selective eCO_2_R to CH_4_ using ten single‐atom transition metals (Sc, Ti, V, Cr, Mn, Fe, Co, Ni, Cu, and Zn) on a B_5_N_3_ monolayer, and favorable screening eCO_2_RR pathways with spin‐polarized computer models.^[^ ^179^ ^]^ Due to hybrid orbitals at the Fermi level caused by doping, single‐atom Ni anchored to a monolayer of B_5_N_3_ demonstrates good stability and conductivity. In this eCO_2_RR to methane, this catalyst shows high theoretical selectivity for CH_4_ and a low limiting potential (−0.21 V vs RHE). Using a facile method, Hussain et al. designed a Cu_2_O‐MoS_2_ composite for eCO_2_RR.^[^ ^180^ ^]^ A reducing current density of 113 mA cm^−2^ is obtained, which is an eightfold increase over bare Cu_2_O (61 mA cm^−2^), and a fourfold increase over MoS_2_ sheets (21.3 mA cm^−2^). Polycrystalline Cu NPs (called Cu‐s) with rich high‐index facets, derived from Cu_2−x_S were prepared via desulphurization and surface reconstruction, presenting an excellent way for investigating the role of surface in electrocatalytic CO_2_ conversion.^[^ ^181^ ^]^ The high CO_2_ conversion performance achieved by the Cu‐s catalyst in H‐cell with 68.6% FE and 40.8 mA cm^−2^ partial current density is a result of the surface reconstruction process in Cu‐s, which creates an increased rate of high‐index facets. Kanase et al. altered the morphology of Cu electrocatalysts by developing Cu_80_Al_20_ alloys on carbon paper.^[^ ^182^ ^]^ The Cu_80_Al_20_, and etched Cu_80_Al_20_ layers are adapted to investigate the fundamental electrochemical characteristics for electrochemical eCO_2_RR using a flow electrolyzer with GDEs in a 1 m KHCO_3_ electrolyte (**Figure** **18**a,b).

Li et al. developed a molecular tuning method for functionalizing electrocatalyst surfaces to stabilize the intermediates for more selective eCO_2_RR to ethylene conversion.^[^ ^183^ ^]^ The attached molecules increase the stability of an intermediate bound to a Cu atom (i.e., bound to a top‐bound CO molecule), enabling ethylene reduction. In a neutral medium, the CO_2_RR to ethylene conversion yields a FE of 72% at 230 mA cm^−2^ partial current density. A 20% energy efficiency and 190 h of steady ethylene electrosynthesis are achieved. Molecular strategies that utilize local molecular tuning will be able to complement heterogeneous catalysts. Metalloporphyrins are used as molecular catalysts in eCO_2_RR to produce CO with high selectivity.^[^ ^184^ ^]^ Many functionalization techniques have been developed on metalloporphyrins to improve eCO_2_RR performance since the first publication on Fe‐TPP (TPP = 5,10,15,20‐tetraphenylporphyrin).^[^ ^185^ ^]^ Immobilizing the porphyrin with carbon enables the dispersion of its catalytic activity in the metalloporphyrin, and prevents the aggregation of metallic active centers in the metalloporphyrin.^[^ ^186^ ^]^ Yan et al. advocated embedding M‐TCPP [M = FeCl, Co, and Ni; TCPP = tetrakis(4‐carboxyphenyl)porphyrin] within Cu‐MOF pores to give additional CO intermediates to the Cu sites, resulting in better CO_2_ to C_2_H_4_ conversion.^[^ ^187^ ^]^ Cu‐MOFs in these composites may effectively protect M‐TCPP and allow CO_2_ conversion, supplying more CO to the Cu sites and contributing to Cu‐MOF‐catalyzed C—C coupling.^[^ ^188^ ^]^ The large active sites of porous electrocatalysts make them excellent for mass diffusion, chemisorption, and intermediate stabilization. CO provided by M‐TCPP around Cu sites might decrease C—C coupling reaction barriers, encouraging the production of C_2+_ products. Therefore, both the FE and the overpotential of C_2_H_4_ are increased as a consequence.

#### 3.2.3 Reduction of CO_2_ to Formate

**Figure 19:**
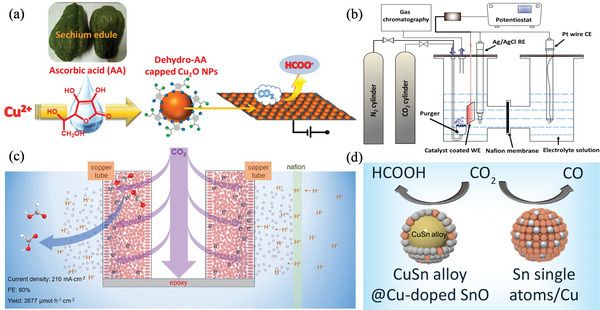
a) Using bioanalytes as Cu_2_O NPs in eCO_2_RR to formate. b) Experimental setup for eCO2RR using two‐electrode systems with N_2_‐purged and CO_2_‐saturated environments in an H‐type electrochemical cell. Reproduced with permission.^[^ ^191^ ^]^ Copyright 2021, Elsevier. c) Copper hollow fiber electrode for efficient eCO_2_RR. Reproduced with permission.^[^ ^192^ ^]^ Copyright 2021, Elsevier. d) Tunable selectivity for eCO_2_RR by bimetallic Cu—Sn catalysts. Reproduced with permission.^[^ ^193^ ^]^ Copyright 2021, American Chemical Society.

Formate produced by eCO_2_RR has fueled indirect formic acid fuel cells and precision chemical synthesis. Pd NPs have been shown to convert eCO_2_RR to formate with considerable efficiency. During electrocatalysis, Pd NPs are deactivated by CO surface poisoning. Unlike Pd, Cu protects it from carbon monoxide poisoning. Du et al. have designed a new Cu‐anchored on hollow carbon sphere catalyst (HCS/Cu‐x, where x represents the mass of CuCl_2_ added in the system), allowing for controllable Cu/C heterogeneous interfaces.^[^ ^189^ ^]^ The optimized HCS/Cu‐0.12 catalyst with a rich Cu/C heterogeneous interface and hollow structure is advantageous to mass transmission. Wu and colleagues devised a novel core‐shell configuration for bimetallic alloy/oxide nanowire catalysts.^[^ ^190^ ^]^ Typically, a core of CuSn alloy ensures high electrical conductivity, while the SnO_2_ shell amorphized with Cu guarantees catalytic activity and selectivity. Molecular dynamics studies further demonstrate that Cu‐doped SnO_2_ layers play a major role in the electrocatalytic selectivity for formate and the control of hydrogen production during electrocatalysis. Using Sechium edule fruit compounds as bioactive compounds, Chowdhury et al. synthesized Cu_2_O NPs (**Figure** **19**a).^[^ ^191^ ^]^ By using modified electrodes, charge transfer resistance may be decreased by as much as 50‐folds, and eCO_2_RR to HCOO may occur in 0.5 m KHCO_3_ electrolyte with a FE of approximately 65–66% within 60 min as the existence of dominant Cu_2_O (111) NPs may explain the selective formation of formate using an H‐type glass reactor (Figure 19b). Zhang et al. studied phase‐inversion/sintering process by constructing a copper hollow fiber for gas‐diffusion electrodes, thus delivering a high FE (80%) at high current density (210 mA cm^−2^) that are 16 to 30 times higher than those of Cu foam and Cu foil, respectively (Figure 19c).^[^ ^192^ ^]^ Zhang et al. designed Cu—Sn composite catalysts for eCO_2_RR, and it was reported that a change in Cu/Sn composition could change the reduction products selectively from formate to CO (Figure 19d).^[^ ^193^ ^]^ The Cu_1_Sn_1_ catalyst has a FE of 95.4% for formate at 1.2 V when it has a CuSn alloy core and a SnO shell structure doped with a minor quantity of Cu. Cu_20_Sn_1_, on the other hand, has a maximum FE of 95.3% at 1.0 V. The introduction of modest quantities of Cu or Sn single atoms in these two catalysts led to a considerable decrease in the reaction free energy, resulting in the synthesis of formate and CO, respectively.

S‐doping and Cu‐alloying can be used to regulate the optimal electronic structure of the Sn active site to favor formate formation while suppressing the CO and H_2_ pathways. Wang et al. synthesized ultrathin Cu_2_SnS_3_ NSs as catalysts and realized a high selectivity and activity for CO_2_RR to formate in the wide potential range from −0.6 to −1.1 V.^[^ ^194^ ^]^ Cu_2_SnS_3_ NSs can be transformed to the CuS@SnO_2_ and Cu_2_O@SnO_2_ through in situ electroreduction. DFT calculations reveal that the electron of Sn^4+^ tends to delocalize and donate to Cu^+^ via the O atom by forming a heterojunction interface between SnO_2_ and CuS/Cu_2_O. The delocalized Sn sites can enhance the affinity for the HCOO* and promote the dissociation of H_2_O to form H* and stabilize the active Sn^4+^ sites for resisting the applied negative potential, thereby improving the activity and selectivity for CO_2_RR in a wide potential window.^[^ ^195^ ^]^

Electrodeposited Bi catalysts were electrodeposited on Cu foams by Li et al. to study the influence of Cu substrates on electrochemical performance.^[^ ^196^ ^]^ Cu not only acts as electrode substrates but also serves as the material actively reducing CO_2_, resulting in Bi/Cu electrocatalysts varied in their morphology and composition. The optimized Bi/Cu materials thus achieve a high activity of 59.7 mA cm^−2^ and a high selectivity of 95%. Li et al. prepared Cu/Bi aerogels with enhanced eCO_2_RR activity using a simple one‐step assembly method.^[^ ^197^ ^]^ At a potential of −0.9 V versus RHE, the Cu_1_Bi_2_ catalyst exhibits excellent eCO_2_RR activity with a FE of 96.57% towards HCOOH, and the FE of HCOOH remains over 80.18% over a wide potential range (−0.8 to −1.2 V vs RHE). The increased eCO_2_RR activity is due to the self‐supporting structure and synergistic effect of Cu and Bi.

**Figure 20:**
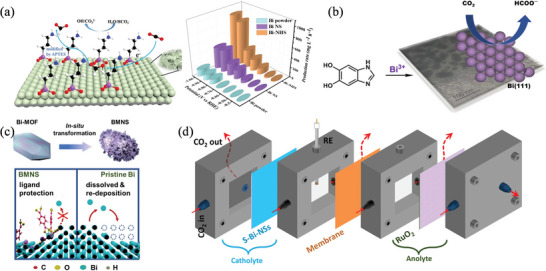
a) eCO_2_R with bismuth NSs decorated with 3‐aminopropyltriethoxysilane. Reproduced with permission.^[^ ^206^ ^]^ Copyright 2021, Published by Elsevier. b) Bismuth MOF for eCO_2_R to formate. Reproduced with permission.^[^ ^207^ ^]^ Copyright 2022, Elsevier. c) Bismuth NSs formed in situ for efficient CO_2_ conversion. Reproduced with permission.^[^ ^208^ ^]^ Copyright 2021, Elsevier. d) eCO_2_RR performance of S‐Bi‐NSs in a flow cell. Reproduced with permission.^[^ ^211^ ^]^ Copyright 2021, American Chemical Society.

Using underpotential deposition, Takashima and colleagues created Pd NPs with Cu atom layers.^[^ ^198^ ^]^ The bimetallic Pd/Cu catalyst shows a higher FE (84%) towards formate than Pd catalysts. The reason is that Pd could transfer charge transfer to Cu, which shifts the average d‐band center of the catalyst downward relative to the Fermi level. Surface‐active sites in porous nanostructures are richer than in bulk materials, which could enhance the catalytic activity. A 3D hierarchical porous structure is constructed using electrochemical deposition strategy which is employed in eCO_2_RR to achieve a high current density of approximately 60 mA cm^−2^.^[^ ^199^ ^]^ In a recent study, Wang et al. manufactured ultrathin ZnIn_2_S_4_ NSs with Zn vacancies to electrochemically reduce CO_2_ to formate.^[^ ^200^ ^]^ Experimental and theoretical results indicate that the Zn‐vacancy‐rich ultrathin ZnIn_2_S_4_ NSs with a high electrochemically active surface area contribute to the enhanced selectivity and activity through optimizing the intermediate binding energy. Lu et al. found that In—N—C catalyst provided a highly efficient method for producing formic acid/formate in aqueous media, with a high TOF of 26 771 h^−1^ at −0.99 V versus RHE.^[^ ^201^ ^]^ Based on DFT calculations, the formation of ^*^OCHO intermediate shows a lower energy barrier on In—N—C catalyst, contributing to the high efficiency of formate formation. Shin et al. described the preparation of hierarchical mesoporous In nanocrystals (NCs) from nanobelts using hydrogen bubbles as geometric templates.^[^ ^202^ ^]^ The as‐prepared catalyst has a large surface area and rich active sites, thus enhancing the eCO_2_RR. The DFT calculations prove that catalytic activity is plane dependent and indicates that it is selective for formate production.^[^ ^199^, ^203^ ^]^ These findings provide fresh insight into the simple fabrication of porous hierarchical nanostructures for selective eCO_2_RR and a high‐performance CO_2_‐to‐formate electrocatalyst. Using in situ electrochemical transformations of (BiO)_2_CO_3_ nanostructures, Peng et al. developed an extremely active and selective Bi‐NSs assembly, which reached nearly 94% FE at −1.03 V (vs RHE) and stable selectivity (>90%) within a large potential window ranging from −0.83 to −1.18 V (vs RHE), as well as excellent durability of 12 h towards formate.^[^ ^204^ ^]^ An edible sponge‐like Bi_2_O_3_ with unique porous morphology and low crystallinity was synthesized, which was in situ reconstructed into 2D NSs containing metallic Bi and Bi_2_O_2_CO_3_, and served as the active species for eCO_2_RR to formate.^[^ ^205^ ^]^ Wang et al. examined the reaction performance and intrinsic properties of functionalized Bi nanosheets (Bi‐NHS) electrocatalysts by adding 3‐aminopropyltriethoxysilane (**Figure** **20**a).^[^ ^206^ ^]^ Experimental results and DFT calculations show that eCO_2_RR to formate on Bi(001)‐NHS surfaces are more energetically favorable than on bare Bi(001) surfaces, and 3‐aminopropyltriethoxysilane are excellent ligands to stabilize the catalytic activities of metallic Bi. Ávila‐Bolívar et al. reported the preparation of a simple, tunable Bi‐based electrocatalyst using MOF as an affordable precursor (Figure 20b).^[^ ^207^ ^]^ The carbon‐rich ligand within this MOF is 1H‐benzo[d]imidazole‐5,6‐diol, combined with bismuth chloride. In theoretical studies, the enhanced eCO_2_RR to formate is linked to metallic Bi sites. Recently, Li et al. obtained ligand‐stabilized Bi NSs which showed remarkable efficiency for eCO_2_RR (Figure 20c).^[^ ^208^ ^]^ 98% FE of formate and excellent durability for 40 h are achieved, which is due to the number of under‐coordinated Bi active sites maintained by residual organic ligands. Xia's group employed Bi‐based MOFs to prepare Bi_2_O_3_@C for high‐efficiency eCO_2_RR.^[^ ^209^ ^]^ The resultant Bi_2_O_3_@C‐800 exhibits a small onset potential of −0.28 V versus RHE, stable FE of 93%, and high partial current density of over 200 mA cm^−2^ at −1.1 V versus RHE for the fast formate production in a flow cell configuration. Electrochemical results demonstrate that the Bi_2_O_3_@C hybrid synergistically promotes selective and fast CO_2_ reduction, where the carbon matrix would help enhance the activity and current density, while the oxides are beneficial for improving the reaction kinetics and selectivity.^[^ ^210^ ^]^ This work provides effective Bi‐based MOF derivatives for the efficient production of formate and offers valuable insights into promoting the rapid and selective CO_2_ reduction technology. Peng et al. created an active and selective hydrangea‐like micro/nanoreactor of ultrathin Bi NSs via in situ electrochemical topotactic production of hierarchical BiOCOOH, which could be used as a micro/nanoreactor for boosting electrochemical activity.^[^ ^211^ ^]^ The material exhibits exceptional electrocatalytic performance for the reduction of CO_2_ to formate, with near‐uniform Faradaic selectivity (>95%) throughout a broad potential range of 0.78 to 1.18 V. Furthermore, without losing the selectivity of a flow cell reactor, this micro/nanoreactor produces significant current densities of about 300 mA cm^−2^ at low applied potentials (Figure 20d). The combination of oxygen‐vacancy‐rich Bi subcarbonate with reduced graphene oxide (Vo‐BOC/G) has been designed for eCO_2_RR‐to‐formate conversion.^[^ ^212^ ^]^ Using 0.1 m KHCO_3_, the Vo‐BOC/G shows 100% formate selectivity at −1.2 V versus RHE and boasts a partial current density of 38 mA cm^−2^. It has been shown that the abundant *V*_o_ defects lower the energy barrier for ^*^CO_2_ formation, which results in high formate selectivity.

**Figure 21:**
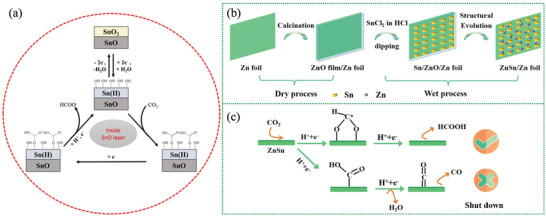
a) Enabling durable selectivity of eCO_2_RR to formate achieved by a multi‐layer SnO_x_ structure. Reproduced with permission.^[^ ^218^ ^]^ Copyright 2021, Elsevier. b) The schematic illustration of ZnSn catalysts growth on Zn foil and c) the possible reaction pathways for the eCO_2_R to C1 products on the surface of ZnSn catalyst. Reproduced with permission.^[^ ^219^ ^]^ Copyright 2021, Elsevier.

VÁvila‐Bolívar et al. prepared a series of Bi, Sn, and Sb/carbon composites for eCO_2_RR, and demonstrated the tri‐metallic Bi—Sn—Sb electrodes exhibited the optimum activity and selectivity towards formate.^[^ ^213^ ^]^ In particular, the Bi_95_Sb_05_/C and Bi_80_Sn_10_Sb_10_/C electrodes maintain high formate efficiency of over 50% after 24 h. A simple fast‐reduction method was used by Zhang et al. to prepare Cu‐decorated Bi/Bi_2_O_3_ nanofoam, which showed excellent electrocatalytic performance towards eCO_2_RR.^[^ ^214^ ^]^ The excellent performance is further ascribed to the substantial microstructural and electronic changes upon the introduction of Cu. Sui et al. prepared bilayers of Bi_2_S_3_ and Bi_2_O_3_ and demonstrated that they enhanced eCO_2_RR performance, delivering 90% formate FE in a wide potential window.^[^ ^215^ ^]^ An essential factor for excellent electrocatalytic activity is the fast transfer of charge at the Bi_2_S_3_/Bi_2_O_3_ interface, the increased number of active sites, and the enhanced CO_2_ adsorption ability. Using cathodically in situ reconstruction, Zhao et al. created ultralong and thin Bi‐organic hybrid nanobelts (Bi‐NBs), which have better eCO_2_RR performances than their discrete counterparts.^[^ ^216^ ^]^ Calculations demonstrate that the high edge‐to‐face ratio of Bi‐NBs is responsible for the enhanced performance, as the rich edge sites aid in the stability of the crucial intermediate ^*^OCHO utilized in formate synthesis. Ning et al. found the SnO_2_ nanoparticles grown onto carbon fiber cloth (SnO_2_/CF) heterojunctions displayed good electrocatalytic activity towards eCO_2_RR, reaching a high FE of 93% and a partial current density of 28.7 mA cm^−2^ in an H‐type cell. The excellent electrocatalytic performances are due to the rebuilding of SnO_2_/CF heterojunctions into SnO_2_/Sn Mott‐Schottky junctions during eCO_2_RR electrolysis.^[^ ^217^ ^]^ DFT revealed that compared to pristine SnO_2_ and Sn, the SnO_2_/Sn heterostructures generated in situ during eCO_2_R helped decreased the energy barrier for formate synthesis. Pan et al. created a multilayer SnO_x_ structure utilizing a ligand‐confined growth approach, which achieved a high formate FE of 93.2% and remained robust for a minimum of five cycles over 40 h electrocatalysis at 1.15 V versus RHE (**Figure** **21**a).^[^ ^218^ ^]^ The multi‐layer structure can stabilize Sn^2+^ species, allowing for very stable formate selection. Li et al. created ZnSn catalysts supported on Zn foil by calcining the pretreatment foils using a mix of dry, wet, and galvanic replacement procedures (Figure 21b).^[^ ^219^ ^]^ Figure 21c depicts the anticipated eCO_2_RR mechanism in this system. Zn centers on Zn foil and ZnO electrodes create CO by eCO_2_RR through a COOH^*^ intermediate route. ZnSn, on the other hand, is formate‐selective in the same way as monometallic Sn. The ^*^O atoms from CO_2_ molecules tend to be absorbed by the oxyphilic surface of Sn, stabilizing the ^*^OCHO intermediates. The ^*^OCHO route will be followed by the ZnSn electrode, culminating in the synthesis of formate. The CO generation route will be shut down due to the deactivation of Zn sites and the decrease in the number of Zn active sites, thus promoting formate synthesis.

Modification of surfaces by amine molecules is a significant way to improve the eCO_2_RR efficiency.^[^ ^220^ ^]^ Exploiting the reduced energy barriers for the protonation of CO into ^*^CHO species stabilized by the —NH_3_^+^ group of zwitterionic glycine,^[^ ^220a^ ^]^ can greatly improve the selectivity of hydrocarbons for Cu electrodes. In a similar way, polyacrylamide was coated on the surface of the Cu electrode to contribute to the electron enrichment of the Cu electrode, which promotes CO dimerization.^[^ ^220b^ ^]^ As a result of hydrogen bonding, the —NH_2_ group ensures CO dimer stability, which significantly influences selectivity regulation.^[^ ^220f^ ^]^ When alkyl chains are lengthened for linear amines (propylamine, hexylamine, oleylamine), CO selectivity improves, but branching polyethyleneimine (PEI) completely blocks the CO route. Pyridinium additives and amine‐containing compounds were also used to increase selectivity.^[^ ^183^, ^221^ ^]^ In bicarbonate solutions, N‐aryl pyridinium electrochemically coupled to polycrystalline Cu produces an N‐substituted tetrahydro‐4,4′‐bipyridine layer, which produces about 80% selectivity for ethylene, ethanol, and propanol.^[^ ^221^ ^]^

### 3.3 Reduction of C=C/C≡C Bond

Electrochemical reduction of unactivated C=C double bonds is difficult because of their large reduction potentials (absolute values). Electron‐withdrawing groups can replace double bonds to make cathodic reductions possible.^[^ ^222^ ^]^ It is possible to formally hydrogenate electron‐deficient olefins in the presence of a hydrogen donor.^[^ ^223^ ^]^ Navarro and colleagues electrochemically reduced Michael acceptors and dienes using Ni— or Fe‐based mediators.^[^ ^224^ ^]^ Tajima and colleagues developed an electrochemical method to reduce Michael acceptors using a polymer‐supported acid.^[^ ^225^ ^]^ In addition, indirect cathodic reductions of C=C double bonds have been documented.^[^ ^224^, ^226^ ^]^ However, all of these techniques use oxidative activation to activate reactants or catalysts, and it is still an unexplored area of research to create electroreductive methods to difunctionalize alkenes.^[^ ^227^ ^]^

**Figure 22:**
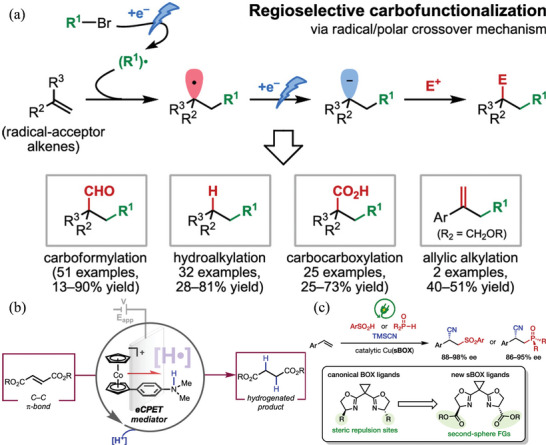
a) Electroreductive carbofunctionalization of alkenes with alkyl bromides via a radical‐polar crossover mechanism. Reproduced with permission.^[^ ^232^ ^]^ Copyright 2020, American Chemical Society. b) Electrocatalytic reduction of C—C *π*‐bonds via a cobaltocene‐derived concerted proton–electron transfer mediator. Reproduced with permission.^[^ ^234^ ^]^ Copyright 2021, American Chemical Society. c) Bisoxazoline ligands enable enantioselective electrocatalytic cyanofunctionalization of vinylarenes. with permission.^[^ ^235^ ^]^ Copyright 2019, American Chemical Society.

C‐heteroatom bonds (e.g., C—O, C—N, C—S, C—Cl) are the most commonly produced by electrochemical methods. C—C bonds nevertheless rarely form (i.e., carbofunctionalization) as it relies heavily on nucleophiles that are prefunctionalized. By using radical precursors such as Langlois reagent (CF_3_SO_2_Na) and 1,3‐dicarbonyl compounds, carbofunctionalization of alkenes has been successfully achieved.^[^ ^228^ ^]^ Xu has also discussed the anodic oxidation of styrenes to radical cations that occurs during the hydroxyalkynylation process.^[^ ^229^ ^]^ Anodically coupled electrolysis has recently been used for the chlorotrifluoromethylation of alkenes and chloroalkylations of alkenes.^[^ ^230^ ^]^ For instance, novel radical cation Diels–Alder reactions by electrocatalysis have now been developed that utilize nonconjugated alkenes as dienophiles.^[^ ^231^ ^]^ Zhang et al. take electrochemistry to the next level by reductively functionalizing alkenes (**Figure** **22**a).^[^ ^232^ ^]^ By selecting the suitable reagents and reaction conditions, a radical‐polar crossover pathway can be established by adding two different electrophiles across an alkene in a highly chemo‐ and regioselective manner. Electroreductive synthesis of alkyl radicals and carbanion intermediates to achieve intermolecular carboformylation, anti‐Markovnikov hydroalkylation, and carbocarboxylation of alkenes‐reactions are obtained with no precedent in the literature. In addition to using readily available starting materials (alkyl halides, alkenes, etc.) and simple, transition‐metal‐free conditions, these reactions exhibit a broad substrate scope and good functional group tolerance. Through electrochemical method, Song et al. seamlessly combined two canonical radical reactions, copper‐mediated radical cyanation, and cobalt‐mediated radical HAT, to increase hydrocyanation enantioselectivity.^[^ ^233^ ^]^ By controlling the potential of electrochemistry, the chemoselectivity of challenging substrates can also be improved. Computer simulations offer insight into the mechanism of enantio‐induction, which comprises chiral catalysts imparting attractive and repulsive non‐covalent interactions to drive the formation of an enantio‐determining C—CN bond. Derosa et al. used a concerted proton‐electron transfer (CPET) mediator consisting of cobaltocene with a Bronsted base to achieve selective hydrogenation of the C—C *π*‐bond in fumarate esters by electrocatalysis (Figure 22b).^[^ ^234^ ^]^ In the presence of the mediator, electrocatalytic hydrogenation shows high selectivity. An analysis of the mechanics reveals two distinct kinetic regimes based on the substrate concentration: at low fumarate concentrations, CPET follows electron‐transfer/proton‐transfer (ET/PT) while at high concentrations, CPET follows ET/PT. A highly enantioselective electrochemical method for the cyanophosphinoylation of vinylarenes has been developed by Fu et al.(Figure 22c).^[^ ^235^ ^]^ This led to the identification of chiral bisoxazolines derived from serine that had ancillary coordination sites as ideal ligands.

By using alkyl bromides, Zhang et al. described electro‐reductive carbofunctionalization of alkenes.^[^ ^232^ ^]^ By adding an alkyl or a CHO/CO_2_H group and the anti‐Markovnikov hydroalkylation process, an alkene can be carboformylated or carbocarboxylated regioselectively. In an electrochemical‐chemical‐like process (ECEC), the cathode undergoes two reduction processes that yield carbanion and radical intermediates, resulting in radical‐polar crossover reactions. Wang et al. generated N‐cyclopropylaniline radical cations electrochemically. Using a home‐built electrochemistry/mass spectrometry platform, a new redox neutral reaction of intermolecular [3+2] annulation of N‐cyclopropylanilines and alkenes is employed to obtain an aniline‐substitute.^[^ ^236^ ^]^ A chain mechanism including radical regeneration and the creation of the neutral product can enhance such a redox‐neutral annulation reaction. In addition, methyl sulfone dimerizations have been documented.^[^ ^237^ ^]^ Electrochemical conditions yield better electrohydrocyclization products than chemical processes using SmI_2_ as the reductant.^[^ ^238^ ^]^ SmI_2_ was also used as a reducer in Handy and colleagues' study, whereas electrochemical conditions yielded only cinnamic acid esters.^[^ ^239^ ^]^ Kise et al. discovered that cinnamic acid esters dimerize stereoselectively.^[^ ^240^ ^]^ Although initial attempts with chiral auxiliaries derived from menthol and borneol yielded little stereoselectivity, a bulky chiral auxiliary readily synthesized from (1R)‐(+)‐camphor proved to be effective; the dimerized product was obtained in a good yield with 92% e.e. upon cleavage of the auxiliary. In the design of catalysts, CPET stages improve reaction efficiency.^[^ ^241^ ^]^ Carbonyl groups are attractive reductive CPET techniques^[^ ^242^ ^]^ like reducing unsaturated substrates.^[^ ^243^ ^]^ Due to the significant structural rearrangement (sp^2^‐to‐sp^3^ hybridization) associated with CPET to a C—C *π*‐bond, reductive CPET with C—C *π*‐bonds remains a popular synthesis option for chemical synthesis.^[^ ^244^ ^]^ Epstein and Flowers conducted mechanistic studies on the application of SmI_2_.H_2_O to the reduction of anthracene or enamines.^[^ ^245^ ^]^ A catalytic approach using C—C *π*‐bond CPET of metal–hydride (M—H) intermediates to C—C bonds^[^ ^246^ ^]^ has been developed when the active catalyst is regenerated with stoichiometric silanes or H_2_.^[^ ^247^ ^]^

**Figure 23:**
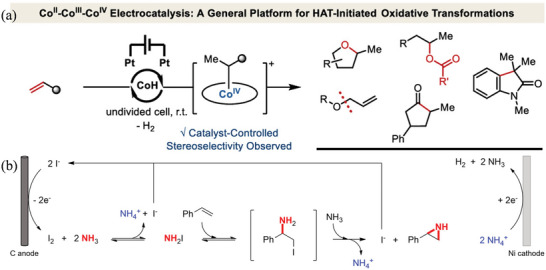
a) Electrocatalytic oxidative hydrofunctionalization reactions of alkenes via Co(II/III/IV) cycle. Reproduced with permission.^[^ ^248^ ^]^ Copyright 2022, American Chemical Society. b) Proposed reaction mechanism for the electrocatalytic aziridination of styrene. Reproduced with permission.^[^ ^249^ ^]^ Copyright 2021, American Chemical Society.

Using Co(II/III/IV) electrocatalysis, Yang et al. described an electrocatalytic platform for oxidative hydrofunctionalization reactions (**Figure** **23**a).^[^ ^248^ ^]^ A set of oxidative hydrofunctionalization reactions are demonstrated via HAT without using a stochiometric chemical oxidant. In addition to hydroalkoxylations, hydroacyloxylations, hydroarylations, semipinacol rearrangements, and deallylations are also included. Mechanical and stereochemical studies support the electrochemical generation of organocobalt(IV) intermediate by an ECEC process. Using ammonia (NH_3_) as an atom‐efficient nitrogen source, Vanhoof et al. reported simple aromatic alkenes' electrocatalytic N—H aziridination, and up to 98% yields were obtained using a graphite anode and Ni cathode, with H_2_ as the only byproduct.^[^ ^249^ ^]^ Iodide is necessary as a redox mediator. The interactions anodically form I_2_ and NH_3,_ creating NH_2_I as a reactive species in aziridination. A reaction mechanism is proposed in Figure 23b. At the anode, I^−^ is oxidized to I_2_, resulting in the reactive species NH_2_I, which is formed by reacting with NH_3_. The addition of vicinal iodoamine to styrene causes it to undergo cyclization toward 2‐phenylaziridine, resulting in the adsorption of vicinal iodide on styrene. Then I^−^ is released, which can be oxidized again. A redox cycle is closed with the reduction of the produced NH_4_^+^ to NH_3_ and H_2_. Water itself can also be reduced to H_2_ and OH^−^, though this is not equivalent.

The CH_3_CN‐involved electrochemical borylation of alkenes and HBpin with no metal catalysts was reported by Zhang et al.^[^ ^250^ ^]^ The site selectivity is achieved by regulating the proportion of HBpin to achieve mono‐ or difunctional borylation of unsaturated bonds. Moreover, the success of gram‐scale experiments and the versatility of conversions confirm the potential applications of this strategy in industrial synthesis. In the mechanism study, N,N‐diisopropylethylamine is revealed to play an essential role in the electrolysis of acetonitrile. Due to difficulties in conventional electrode‐mediated reductions, such as competing HER and substrate polymerization routes, electrocatalytic C—C *π*‐bond reduction (eCPET) might be a better option.^[^ ^3a^ ^]^ The Chalkley group^[^ ^234^ ^]^ investigated the possibility of transferring a stored hydrogen atom to an electrode and an acid solution.^[^ ^251^ ^]^ Thus, the researchers formulate the cobaltocenium redox mediator incorporating N,N‐dimethylaniline as a Bronsted base ([CpCoCpNMe_2_]‐[OTf]) to dissociate the redox and protonation sites and minimize unwanted HER reactions.^[^ ^252^ ^]^ These reductions can also be applied to olefins with an allylic group. Duach and colleagues demonstrated electrochemical deprotection of allyl carbamates, and it was reported that the allyl group could be readily removed by electrogenerated low‐valent Ni species.^[^ ^253^ ^]^ This method has also been used for the deprotection of allyl carbonates.^[^ ^254^ ^]^ Hudlicky et al. determined that cinnamyl groups have a lower reduction potential than primary allyl groups, which eliminated the need for a Ni mediator to reduce cinnamyl ethers.^[^ ^255^ ^]^ However, a mercury cathode is required because of the high reduction potential (absolute value). Under electrochemical circumstances, a cinnamyl group in a molecule is favored to break, whereas an allyl group is unchanged. Through the electrochemical reduction of Michael acceptors, the electroreductive cyclization technique may be utilized to create intramolecular reductive coupling with aldehydes or ketones. Little and colleagues assumed that electrons pass from the Ni^1+^complex to the C=C double bond through an inner sphere mechanism, based on the fact that substitution at the iminyl carbon of the sale ligand prevents cyclization.^[^ ^256^ ^]^ A covalent link between the Michael acceptor and the mediator reduced form is projected to develop in this process; homolysis of this bond creates a protonated ‐radical ion, which participates in cyclization after being protonated. This method may also be used to oxidize Michael acceptors intramolecularly as an alternative. Nishiguchi and coworkers employed magnesium as a reductant to carboxyalkylate‐activated olefins like styrenes or Michael acceptors.^[^ ^257^ ^]^ The same group also reported cathode‐reducing electron‐deficient olefins to radical anions by adding two equivalents of acyl chloride/acid anhydride or N‐acyl imidazole after radical anions are reduced to vicinal bis‐acylation products.^[^ ^227^, ^258^ ^]^

**Figure 24:**
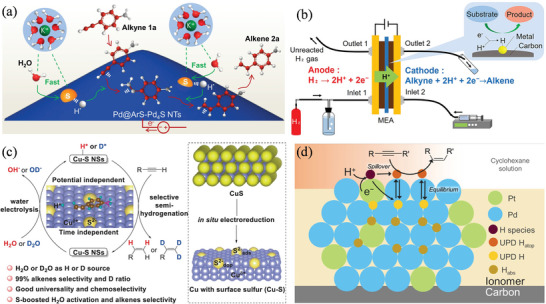
a) An alkyne semihydrogenation reaction mechanism on a Pd@ArS‐Pd_4_S NTs cathode. Reproduced with permission.^[^ ^127f^ ^]^ b) Schematic image of the PEM reactor. Reproduced with permission.^[^ ^264^ ^]^ Copyright 2019, American Chemical Society. c) A schematic illustration of the semi‐hydrogenation strategy. Reproduced under the terms of the Creative Commons Attribution 4.0 International License.^[^ ^124a^ ^]^ Copyright 2021, The Author(s). Published by Springer Nature. d) An electrocatalytic semihydrogenation reaction of alkyne with a Pt—Pd electrocatalyst. Reproduced with permission.^[^ ^265^ ^]^ Copyright 2022, American Chemical Society.

It has also been demonstrated that geminal double carboxylation of imine derivatives occurs.^[^ ^258^ ^]^ In reducing styrene, the phenyl radical anion converts the nucleophilic terminal carbon into a carbonyl radical. Cyclopropyl spirolactone was discovered to be formed from cyclized homoenolates after the loss of a phenol.^[^ ^259^ ^]^ A pilot plant‐scale electrochemical reduction of allyl acetate was also undertaken during the preparation of Ceftibuten. This reaction was shown to function best with a tin cathode, leaving carboxylates, ‐lactams, and sulfoxides untouched.^[^ ^260^ ^]^ Direct electrochemical reduction of aromatic systems may be problematic due to the electron‐rich nature of arenes. In early experiments, however, it was found that electrochemical conditions could facilitate the creation of solvated electrons, thereby facilitating the reduction of aromatic compounds.^[^ ^261^ ^]^ Aqueous solutions can be reduced via comparable processes to arenes in the present circumstances.^[^ ^262^ ^]^ Ishifune and colleagues devised a Birch‐type electron transport mechanism for tBuOH, and an anodic production of Mg^2+^ was thought to mediate electron transport.^[^ ^263^ ^]^ A reference for controlling chlorinated alkene reduction products and preventing pollution from toxic intermediate products formed during incomplete dechlorination was obtained using the stepwise cleavage of chlorinated alkenes on Fe—Ni/rGO/Ni foam during a dichlorination. Semihydrogenation of alkynes is electrocatalyzed by [Ni(bpy)_3_]^2+^, a simple Ni complex found in nature.^[^ ^124b^ ^]^ The electrocatalytic cycle is thought to start atypically with a nickelacyclopropene complex, which is protonated further and transformed into the hypothesized Ni^2+^—vinyl intermediate before the olefin is created, based on the (spectro)electrochemistry. Through homogeneous electrocatalysis, it is possible to enhance the yields and stereoselectivity of alkyne semihydrogenation. Gao et al. found that bulk sulfur anions diminish alkene adsorption, whereas surface thiolates reduced water activation energy and Gibbs free energy for H^*^ production, thus suggesting an S‐tuned effects and reagent concentration adjusting strategy to improve electrocatalytic alkyne semihydrogenation.^[^ ^127f^ ^]^ Self‐supported Pd nanotips with sulfur modifiers were found to produce up to 97% conversion yield, 96% selectivity, 75% flux efficiency, and a reaction rate of 465.6 mmol h^−1^ m^−2^ during electrochemical alkyne semihydrogenation. The excellent performances are due to the high‐curvature structures, which concentrate K^+^ by raising the electric field at their tips, encourage H^*^ generation from water electrolysis via sulfur anion‐hydrated cation networks, and thus improve alkyne conversions. The alkyne semihydrogenation process occurs over Pd@ArS‐Pd_4_S NTs cathodes, as shown in **Figure** **24**a. The reaction begins with the adsorption of 1a and water on the surface of Pd@ArS‐Pd_4_S NTs. H^*^_ads_ is generated from water electroreduction, and then H^*^_ads_ adds to the C≡C bond of a nearby 1a to form the carbon radical intermediate, which abstracts another H^*^_ads_ to produce the alkene product 2a. To hydrogenate alkynes electrocatalytically, Fukazawa et al. used a system with proton‐exchange membranes which produces hydrogenated products without hydrogen gas (Figure 24b).^[^ ^264^ ^]^ At the cathode, protons travel through the polymer and are reduced to monatomic hydrogen species (H_ad_) on the catalyst's surface, which then combines with the substrate to generate the hydrogenated product. The optimal catalyst, according to the reports of Wu et al., should have a low alkene adsorption energy and a higher binding energy with active atomic hydrogen (H^*^) produced by water electrolysis (Figure 24c).^[^ ^124a^ ^]^ By in situ electroreduction of CuS_2_, surface S‐doped and ‐adsorbed Cu nanowire sponges have been produced, with over 99% selectivity over Cu equivalents that lack S. The generation of active H^*^ from water electrolysis is maximized by an S anion‐hydrated cation network (S_2_‐K^+^(H_2_O)_n_) between surface adsorbed S_2_‐ and K^+^ in the KOH electrolyte. Nogami et al. conducted a thorough examination of electrocatalytic hydrogenation of alkynes in a PEM reactor using different Pt‐Pd electrocatalysts.^[^ ^265^ ^]^ Owing to the synergistic effect of Pt—Pd electrocatalysts, the selectivity and production of (Z)‐alkene are significantly enhanced (Figure 24d).

### 3.4 Reductive Coupling Reaction

Recent cost reductions in renewable energy have resulted in the development of electro‐reductive coupling as a viable technology to create higher‐value fuels and chemicals from low‐value carbon‐oxygenates.^[^ ^266^ ^]^ The eCO_2_RR into C—C compounds like ethanol and acetic acid is perhaps the most well‐known example of this upcycling.^[^ ^267^ ^]^ Other organic molecules, such as aldehydes, ketones, and olefins, can be electro‐reductively reacted.^[^ ^268^ ^]^ Biomass can also be used to make these organic molecules, which is crucial for the synthesis of fuels and chemicals with a higher molecular weight. Despite much research focusing on electrochemical reduction for upgrading, electroreductive coupling has received far less attention.^[^ ^269^ ^]^

**Figure 25:**
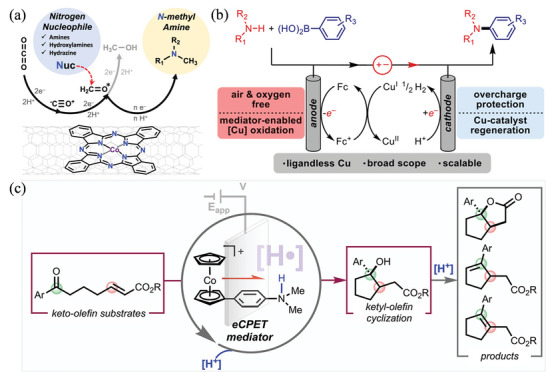
a) Diagram showing the mechanism of electrochemical reductive N‐methylation with CO_2_ catalyzed by CoPc/CNT. Asterisks indicate that a species has been adsorbed on or near the electrode. Reproduced with permission.^[^ ^277^ ^]^ Copyright 2021, American Chemical Society. b) A copper ligandless electrocatalyst for anaerobic Chan–Lam coupling reactions with a mediator. Reproduced with permission.^[^ ^278^ ^]^ Copyright 2021, American Chemical Society. c) A CPET mediator allows electrocatalytic ketyl‐olefin cyclization at a favorable applied bias. Reproduced with permission.^[^ ^279^ ^]^ Copyright 2022, American Chemical Society.

Recently, Chadderdon et al. used distance selective surface poisoning to examine the electrochemical reduction process of furfural on Cu.^[^ ^270^ ^]^ Direct reduction products (alcohol and alkyl compounds) need direct contact with the electrode, whereas furfuryl coupling occurs distant from the electrode. Diaz et al. used an anion exchange membrane flow device to measure the high furfural conversion rate to the hydrodimer.^[^ ^271^ ^]^ It is shown that furfural is converted to the hydrodimer at a high rate, indicating that surface conditions and/or pH play a pivotal role in furfural conversion. Despite these efforts, it remains a mystery how the electroreductive coupling of carbonyls occurs at the molecular level. While it is well‐known that catalysts influence dimerization selectivity, the underlying mechanism remains unknown.^[^ ^268^ ^]^ Because of the well‐established mechanism and relative simplicity, electrochemical benzaldehyde reduction is a suitable model for studying this phenomenon. Interest in benzaldehyde reduction has rekindled since Song et al.^[^ ^272^ ^]^ and following work at Pacific Northwest National Laboratory.^[^ ^273^ ^]^ It is proposed that acid‐catalyzed electron transfer disproportionation can be used for reduction at high pH.^[^ ^274^ ^]^ Recent research suggested that dimers can be made on Co and Cu, though the degree of dimerization is unknown because of interference from C supports.^[^ ^273a^ ^]^ A new mechanism for electrooxidative double C—H arylation was identified by CV, kinetic, and computational studies on cobalt electrocatalysis.^[^ ^275^ ^]^ Contrary to these catalysts, Song et al. suggested only the alcohol was formed for benzaldehyde reduction using Pt group metals (Pt, Pd, Rh, Ni).^[^ ^272^ ^]^ Both Ni and RANEYNi electrodes have been found to show comparable alcohol selectivity.^[^ ^276^ ^]^ Rooney et al. achieved an electrochemical reaction for reductive N‐methylation with CO_2_ and showed compatibility with amines, hydroxylamines, and hydrazine.^[^ ^277^ ^]^ An electrophilic carbon intermediate is formed through the chemical condensation of adsorbed or near‐electrode formaldehyde formed from CO_2_ reduction with nucleophilic nitrogenous reactants, which is reduced in aqueous media by cobalt phthalocyanine molecules supported on CNTs (**Figure** **25**a). Walker et al. showed how to use substoichiometric concentrations of redox mediators to overcome the constraints of Cu‐catalyzed electrosynthesis in Figure 25b.^[^ ^278^ ^]^ Mediators play multiple roles by i) being capable of rapidly oxidizing low‐valent Cu intermediates, ii) regenerating the catalyst by removing the Cu metal from the cathode and exposing the active Pt surface to reduce proton content in the substrate, and iii) protecting the substrate from oxidation due to anodic overcharge, mediators serve multiple purposes. Researchers have recently shown that reductive CPET can be used to transfer a net hydrogen atom to organic materials. However, the effectiveness of reductive CPET in bond formation beyond homocoupling is underdeveloped.

By employing a cross‐cobaltocene base ([CpCoCpNMe_2_][OTf]) as a mediator, Derosa et al. synthesized keto‐olefin substrates that undergo cyclization following ketyl radical generation by eCPET (Figure 25c).^[^ ^279^ ^]^ By examining cis‐lactone and alkene products that are prepared from acetophenone‐derived substrates under the influence of tethered acrylate radical acceptors, the authors demonstrated ketyl‐olefin cyclization. A mixed order in the substrate and acid, as well as a Hammett plot with a modest negative slope illustrating the contribution of sequential CPET and ET/PT steps to the overall reaction rate and indicating O—H bond formation at the outset of the reaction, based on mechanistic analysis of the 2H^+^/2e^−^ process. It is feasible to get ketyl radicals at very moderate reduction potentials via controlled potential electrolysis, allowing functional group tolerance over a wide range of substrates.

**Figure 26:**
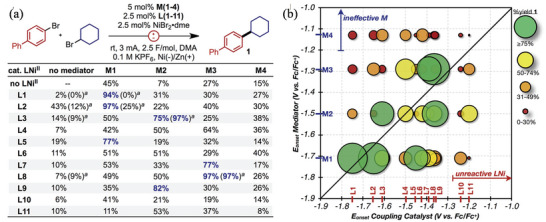
a) Table of GC yields for catalyst–mediator combinations and b) yields plotted as a function of catalyst–mediator *E*_onset_ for electrochemical reactions only. (% yield) a denotes yields from reactions with Zn^0^ powder as the reductant. Reproduced with permission.^[^ ^285^ ^]^ Copyright 2022, American Chemical Society.

Using reactivity and in situ spectroscopic tests, Anibal et al. assessed the electrochemical reduction of benzaldehyde on Pd, Pt, Cu, and Au foils, and discovered that Cu was the best coupling metal among the four.^[^ ^268^ ^]^ There is a ketyl radical intermediate and reduction products on the Au and Cu surfaces, and Cu holds the maximum radical concentration. When unstable benzaldehyde intermediates are decarbonylated on Pd and Pt surfaces, certain quantities of CO poison are produced. According to spectroscopic analysis and reactivity data, various metals' C—C coupling activity for carbonyl species may be assessed based on ketyl radical stability. Molecular scientists have identified Ni‐electrocatalyzed C—H activation to avoid substrate prefunctionalization and chemical oxidation by electricity.^[^ ^280^ ^]^ Under extremely mild circumstances, the vigorous Ni‐electrooxidations allow for plenty of C—C, C—O, and C—N production. Huang et al. showed that by creating in situ redox‐active esters, a moderately reductive Ni‐electrocatalytic system could link two distinct carboxylates, a process known as doubly decarboxylative cross‐coupling.^[^ ^281^ ^]^ This reaction requires no stoichiometric metals or photochemical conditions, accepts a wide variety of functional groups, is scalable, and has been used to synthesize 32 known compounds, cutting the number of stages in the process by 73%. In the resulting electrocatalytic system, aryl, heteroaryl, or vinyl bromides can be reductively coupled with primary or secondary alkyl bromides in a practical, scalable, and versatile way. With the vast differences in yields between coupling reactions with additional redox shuttles (typically >80%) and those without (typically 20%), overcharge prevention becomes increasingly important for electrosynthetic techniques. Research suggested that Ni dimerization occurred in the presence of alcohol as a cosolvent and higher concentrations of benzaldehyde.^[^ ^273a,b^ ^]^ The work by Ang et al. provided a mild and efficient electrochemical thiolation of alkyl bromides with functionalized bench‐stable thiosulfonates in order to obtain alkyl sulfides with excellent product yields and broad tolerance of functional groups.^[^ ^282^ ^]^ A wide variety of substrates can be processed with outstanding yields and shielded from over‐reduction at large currents. A general and practical electro‐reductive Ni‐catalytic system was used by Sun et al. to electrocatalyze the carboxylation of unactivated aryl chlorides and alkyl bromides with CO_2_.^[^ ^283^ ^]^ In addition to aryl bromides, iodides, and sulfonates, electrochemical carboxylations of (pseudo)halides can also undergo this reaction readily without sacrificial electrodes. This process appears to be initiated by the oxidative addition of aryl halides to the Ni° complex, followed by the reduction of the aryl‐Ni^2+^ adduct to the Ni^1+^ species and carboxylation with CO_2_. An enantioselective C(sp^3^)—C(sp^2^)—XEC) catalyzed by Ni was reported in 2019 by Reisman and colleagues, in which they examined an asymmetric electroreductive coupling between alkenyl bromides and benzyl chlorides.^[^ ^284^ ^]^ In general, electro‐reductive cross‐electrophile couplings are not as broad as reactions with chemical reductants, but they are attractive for the direct C—C coupling of electrophiles. To enhance electro‐reductive cross‐electrophile coupling reactions, Zakasee et al. demonstrated mediator‐assisted electrocatalysis.^[^ ^285^ ^]^ All possible pairings that catalyze reactions are summarized in **Figure** **26**a, and as a general rule, mediated reactions yield higher yields than reactions without a mediator. The presence of high‐potential mediators limits reactions, most likely because the mediator prefers to be reduced over the catalyst. In addition, the chance of a catalyst–mediator combination operating for XEC is below −1.3 V and near the diagonal line where the catalyst and mediator onset potentials are equal. The redox potentials of reducing catalysts in this area are high, and they are matched with coupling catalysts. Several empirical insights can be gained from the data presented in Figure 26b that could be used to help select catalysts–mediators in the future. Mismatched combinations that either do not shield the catalyst from over‐reduction (bottom‐right quadrant, *E*_LNi_ ≫ *E*_med_) or impede catalyzing reductive processes (top‐left quadrant, *E*_med_ ≫ *E*_LNi_) are shown by regions on the plot that depart from the 1:1 diagonal.

**Figure 27:**
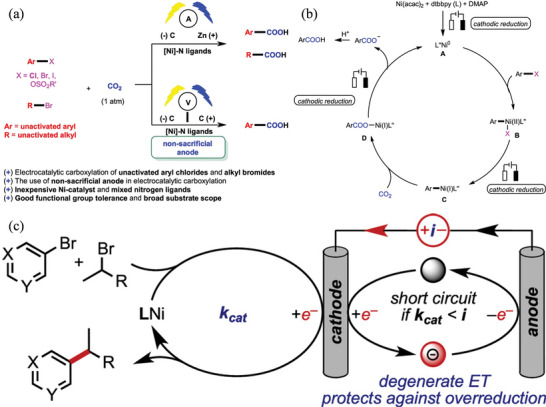
a) Electrocatalytic carboxylating unactivated organohalides with CO_2_. b) Proposed reaction pathway is started up via the electro‐generated Ni^0^ species. Reproduced under the terms of the Creative Commons Attribution 4.0 International License.^[^ ^283^ ^]^ Copyright 2021, The Author(s). Published by Springer Nature. c) Overcharge protection for coupling catalysts with redox cocatalysts enables general XEC reactions. Reproduced with permission.^[^ ^290^ ^]^ Copyright 2020, American Chemical Society.

A bis(oxazoline) ligand derived from aminoindanol was used to obtain excellent enantioselectivity in this process. As coupling agents, N‐hydroxyphthalimide esters were used in the paper published by Bio and colleagues in 2018, which allowed cathodic reduction of the redox active ester in a cathodically coupled electrolysis towards the desired reactivity concurrently with the reduction of the AgICl complex in a cathodic coupled electrolysis.^[^ ^286^ ^]^ The C(sp^3^) radical is intercepted by a Ni catalyst, formed by oxidatively adding electrogenerated Ni^0^ to aryl halide. Ni^3+^ species are reductively eliminated to create the C(sp_2_)—C(sp^3^) coupled product. Anodic oxidation is a typical process to complete electrochemical reactions, which allows electron‐rich tertiary amines to serve as sacrificial reductants. According to a report by Loren's group in 2019, N‐hydroxyphthalimide tetramethyluronium hexafluorophosphate and alkyl carboxylates were used to develop the N‐hydroxyphthalimide esters before electrolysis.^[^ ^287^ ^]^ Gagné proposed Ni^0^–catalyzed reductive coupling of alkyl halides with ‐unsaturated carbonyls.^[^ ^288^ ^]^ In the presence of a Ni^0^–tpy catalyst, alkyl halides (e.g., secondary, tertiary, or sterically hindered primary ones) and electrophilic olefins were reacted. The gram scale reaction produces middle to high yields of compounds (45 to 82%). Through mechanistic analyses, the scientists rule out the possibility that organomanganese species are involved in the catalytic cycle. Through a shielding effect, dimerization is controlled and reductive coupling is achieved, and the silyl‐enol ethers are separated in 82% yield after trapping the intermediate enolate with R_3_SiCl.^[^ ^289^ ^]^ Sun et al. used a generic and practical Ni‐catalytic system to electrochemically carboxylate aryl chlorides and unactivated alkyl bromides with CO_2_ (**Figure** **27**a).^[^ ^283^ ^]^ Unactivated aryl bromide, iodide, and sulfonate molecules can also undergo this process smoothly. Moreover, aryl dihalides were electrochemically carboxylated with CO_2_ without using sacrificial electrodes in a catalytic electrochemical process. A catalytic cycle was offered as a possibility (Figure 27b). The complex Ni(acac)_2_ must be ligated with bipyridine and include DMAP on the cathode to form the L′′Ni^0^ species A. The adduct B is then formed by oxidizing A to the aryl halide, which is then reduced to Ni^1+^ species C by the cathode. When C interacted with CO_2_, the Ni carboxylate intermediate D was produced. The active Ni^0^–catalyst A was recovered, and carboxylate was generated as a result of ligand exchange and reduction of the Ni carboxylate complex, which would then be protonated to provide the desired product. Truesdell et al. devised a mechanically‐driven electrochemical technique for XEC, which took advantage of redox‐active shuttles developed in the energy storage community to protect the electrocatalysts from overreduction (Figure 27c).^[^ ^290^ ^]^ Electrosynthesis requires overcharge prevention since yields tend to be higher with redox shuttles (typically >80%) and lower without (typically 20%).

Combining organometallic reagents with enones to create silyl enol ethers offers flexibility for difunctionalizing activated olefins but using the amount of organometallic reagent required may prove prohibitive. Huihui et al. created silyl enol ether by catalyzing cross‐electrophile couplings of unrestricted primary alkyl bromides with enones and chlorosilanes using a Ni–complexed ortho‐brominated terpyridine ligand.^[^ ^289^ ^]^ Ang et al. demonstrated an efficient and mild electrochemical thiolation of alkyl bromides using functionalized bench‐stable thiosulfonates to produce high‐efficiency and broad‐tolerance alkyl sulfides.^[^ ^282^ ^]^ CV and potentiostatic analysis are used to elucidate the mechanism of this electrocatalytic thiolation process.

## 4 Paired Electrolysis of Organic Molecules

**Scheme 2:**
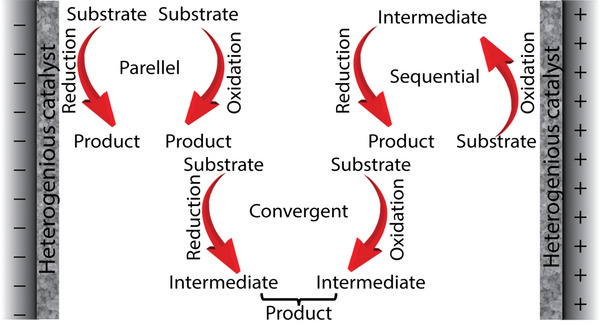
Schematic illustration of electrocatalytic paired heterogeneous catalysis.

Paired electrolysis has received much interest recently because this efficient and atom‐friendly technology has benefited industrial synthesis and energy conversion processes.^[^ ^291^ ^]^ Despite significant advances, strategic utilization of paired electrolysis in organic reactions remains uncommon. Synthetic solutions and novel reaction designs can be recurrently produced through continuous and convergent coupled electrolysis, in which the cathode and anode are used in tandem (**Scheme** **2**). Interelectrode mass transfer may compete with electrically generated reactive intermediates and undesired degradation pathways. In many cases, it is also essential to balance the frequencies of cathodic and anodic events to maintain response selectivity. In this section, we will examine recent breakthroughs that have been achieved through paired electrolysis specifically for addressing synthetic difficulties.

### 4.1 Parallel Paired Electrolysis

**Figure 28:**
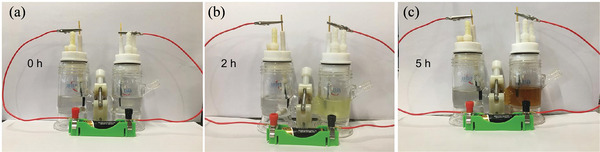
a–c) Time‐dependent photos of a CoP||Ni_2_P electrolyzer setup driven by a 1.5 V battery for both anodic oxidation of octylamine and cathodic reduction of nitrobenzene to azoxybenzene. Reproduced with permission.^[^ ^297^ ^]^ Copyright 2019, Oxford University Press.

Both reduction and oxidation processes occur continuously at electrodes deprived of interfering in parallel paired electrolysis, resulting in reduced and oxidized products at the cathode and anode, respectively.^[^ ^292^ ^]^ Wu et al. studied the relation between the production of hydrogen gas and anodic oxidation processes, which may provide insights into hydrogenolysis and hydrogenation reactions.^[^ ^293^ ^]^ In a paired electrosynthesis cell, NiB_x_@NF was used as both the cathode and anode for the hydrogenation and oxygenation of organic molecules.^[^ ^294^ ^]^ With the concomitant hydrogenation of *p*‐nitrophenol and oxygenation of 5‐HMF, excellent conversion selectivity and efficiency are found. More energy‐efficient coupled electrochemical reactions may be substantially expanded by coupling an electrolysis process with the generation of a chemical substrate or reagent.^[^ ^293^ ^]^ The most crucial process is the Chlor‐alkali procedure, which generates Na and chlorine hydroxide at the cathode and anode.^[^ ^295^ ^]^ eCO_2_RR is coupled with water oxidation at the anode to produce dioxygen. The anodic reaction requires a considerable overpotential and synthesizes dioxygen with low economic value.^[^ ^296^ ^]^ Moeller and Berlinguette's groups recently performed parallel paired electrolysis in split cell systems, demonstrating that significant oxidative mechanisms may be effectively implemented with CO_2_ removal. Amino‐, azo‐, and azoxy‐aromatics were electro‐synthesized using nitroarene feedstocks and a CoP NS cathode by Chong et al.^[^ ^297^ ^]^ Different bias factors produce a wide range of functional group‐tolerant amino‐, azo‐, and azoxy‐ compounds with outstanding selectivity and high yields (**Figure** **28**a–c). Water as the hydrogen source allows for moderate to excellent yields of asymmetric azoxy‐aromatics, which are synthetically substantial and challenging to synthesize. High concentrations of deuterium (deuterated aromatic amines) can be easily produced from D_2_O. The aliphatic amines convert to nitriles during anodic oxidation in conjunction with the CoP||Ni_2_P two‐electrode electrolyzer. Significantly less voltage (1.25 V) is essential to get a current density of 20 mA cm^−2^ for overall water splitting (1.70 V). The corresponding reduction and oxidation reactions can be determined by a 1.5 V battery to produce azoxybenzene and nitrile with high selectivity and yields, demonstrating the method's adaptability.

Co_3_S_4x_ NS is an extremely effective cathode for the specific transition of nitroarenes hydrogenation to respective amino arenes at low overpotential using H_2_O as the source of hydrogen.^[^ ^298^ ^]^ Various amino arenes with functional group compatibility and high selectivity with highly reducible groups (such as C=O, CN, C—I, C=C, C—Br, C=N, and CC) are produced. The gram‐scale production of aminobenzene (0.714 g) at the Co_3_S_4x_ NS cathode and high‐yield dihydro isoquinoline (0.642 g) at the Ni_2_P NS anode are achieved in a Co_3_S_4x_ NS||Ni_2_P two‐electrode cell. Theoretical and experimental findings reveal that sulfur vacancies can promote intrinsic activity and improve selective specific deposition of the nitro group to establish active hydrogen from water electrolysis, resulting in exceptional functional group tolerance and high selectivity for fragile functional groups.

In a split cell, Kubiak and colleagues combined the cathodic synthesis of CO with the anodic fabrication of benzimidazole derivatives.^[^ ^299^ ^]^ In the anodic technique, a Ce facilitator is used to support the oxidative condensation of lignin sawdust‐derived syringaldehyde and 2‐amino aniline. In the cathodic chamber, a Re mediator is utilized to convert CO_2_ to CO. Zha and colleagues have described a coupled system in which Sn‐mediated ketone allylation and halide‐mediated alcohol oxidation occur simultaneously in a split cell.^[^ ^300^ ^]^ Fuchigami and Atobe created a parallel balancing electrolysis system by combining the oxidation of secondary BA with the reduction of benzyl halides using a microflow reactor.^[^ ^301^ ^]^ Two products can result from reducing and oxidizing a common starting material (known as deviating paired electrolysis by Frontana‐Uribe and colleagues).^[^ ^292^ ^]^ De Vos and coworkers, for instance, created a method for synthesizing diol and diacids derivatives from dienes electrolysis in an undivided cell containing CO_2_, trifluoroacetic acid, and trimethylamine.^[^ ^302^ ^]^ In a previous illustration by Atobe, electrogenerated Br^+^ with anodic olefin dibromination was coupled with cathodic epoxidation by O_2_ reduction catalyzed by vanadium.^[^ ^303^ ^]^ Zhang and colleagues have recently reported using a CoP NS cathode or a Ti mesh‐supported Co_3_S_4_ NS with sulfur vacancies to electro synthesize ‐azoxy selectively, azo‐, and amino‐aromatics from nitroarenes.^[^ ^298^ ^]^ A wide range of imprecisely malleable functional groups can be considered acceptable when water is used as the hydrogen source to synthesize the required reductive nitro group products. The observed reactivity is dependent on the selective adsorption of nitro group on these cathodic materials. Effectively replacing the kinetically slow OER process enables the gram‐scale anodic synthesis of octylnitrile from octylamine or dihydroisoquinoline from tetrahydroisoquinoline. When the same material is synthesized in the cathodic and anodic processes, this paired electrolytic method is known as linear paired electrolysis.^[^ ^292^ ^]^ HO^•^ radicals and Fe salts mediate the oxidative decarboxylation of D‐arabinose from sodium gluconate in the catholyte, whereas the anodic reaction is a direct decarboxylation.^[^ ^304^ ^]^ Llorente et al. demonstrated that o‐phenylenediamine and syringaldehyde were oxidized to produce 2‐(3,5‐dimethoxy‐4‐hydroxyphenyl)benzimidazole via a molecular electrocatalyst in the presence of paired electrolysis.^[^ ^299^ ^]^ The electrocatalyst ceric ammonium nitrate facilitates the condensation reaction of syringaldehyde—diamine, which releases protons in the solution. According to Li et al. findings, it is possible to perform simultaneous cathodic reduction of CO_2_ to CO and oxidation of organic substances with increased economic significance than dioxygen.^[^ ^305^ ^]^ During the 3 h of tandem electrolysis at an exterior bias of 0.70 V, 1‐phenylethanol is converted into acetophenone, and CO_2_ is converted into CO.

**Figure 29:**
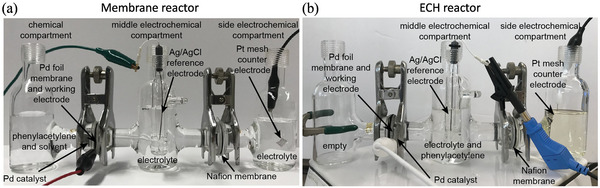
Photographs of the a) membrane and b) ECH reactors. Reproduced with permission.^[^ ^306^ ^]^ Copyright 2019, American Chemical Society.

Sherbo et al. reported that organic reaction chemistry can be conducted without electrolyte contamination in a membrane reactor and organic solvents.^[^ ^306^ ^]^ **Figure** **29**a and Figure 29b depict the images of the membrane and electrochemical hydrogenation reactors. In both instances, the middle and right compartments of the cell contain electrolytes, with a Nafion membrane isolating the oxidative (side compartment) halves and reductive (middle compartment). The organic substrate is dissolved in a suitable solvent inside the membrane reactor's chemical chamber, whereas the Pd black catalyst confronts the side electrochemical chamber. The dissolution of the organic substrate takes place in the electrochemical hydrogenation reactor's middle chamber, where the Pd catalyst comes into contact with the middle chamber. As a result, the chemical compartment of the electrochemical hydrogenation reactor is inoperable.

### 4.2 Sequential Divergent Paired Electrosynthesis

To successfully manufacture the product that is sought after, it is necessary to differentiate convergent and sequential paired electrolysis. Waldvogel recently published a single‐pot electrochemical method for converting aryl aldoximes to aryl nitriles without formal water loss.^[^ ^307^ ^]^ Aldoxime is oxidized at the anode to yield nitrile oxide, which is further reduced at the cathode with a lead electrode to produce aryl nitrile. An article describing the TEMPO‐catalyzed electrosynthesis of N‐heteroaromatic substances from biaryl ketoximes was published in 2018 by Xu and his colleagues.^[^ ^308^ ^]^ In the presence of a base, the action of anodically produced TEMPO^+^ is hypothesized to generate iminoxyl radicals. Before rearomatization, the iminoxyl radical is cyclized onto the phenyl ring, resulting in N‐heteroaromatic N‐oxide. Using a lead cathode, the breakage of the cathodic NO bond is noted, leading to the formation of N‐heteroaromatic compounds (deoxygenated). Previously, the synthesis of biaryl ethers by Nishiyama using oxidative CO dimerization of ortho‐dihalophenols and cathodic reduction was described.^[^ ^309^ ^]^ In light of the relative stability of Ni complexes and the spatial secession of electrolysis, Ni‐catalyzed paired electrolysis is a potential material for advancing hard redox‐neutral cross‐coupling processes.^[^ ^310^ ^]^ Zhu et al. proposed a cross‐coupling reaction catalyzed by Ni under mild reaction conditions by employing concerted paired electrolysis.^[^ ^311^ ^]^ This electrochemical transformation involves the cathodic reduction of Ni^1+^ to Ni^0^ and the anodic oxidation of Ni^2+^ to Ni^3+^, resulting in a cost‐effective and environmentally friendly cross‐coupling procedure. In addition, electrochemistry has the opportunity to drastically modify the oxidation states of catalysts' metal atoms to facilitate elementary reactions. Due to severe reaction conditions, the economically advantageous bond formation reactions of Ni‐catalyzed carbon‐heteroatom have restricted successful applicability in conventional two‐electron mediated cross‐coupling procedures as the formation of the bond requires extremely harsh reaction conditions.

**Figure 30:**
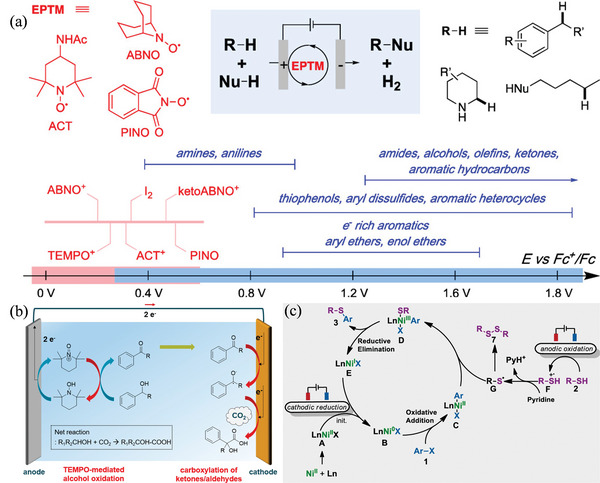
a) Electrochemical oxidation of organic molecules at lower overpotential. Reproduced with permission.^[^ ^313^ ^]^ Copyright 2020, American Chemical Society. b) Electrosynthesis of *α*‐hydroxy acids using alcohols and CO_2_ without sacrificial anodes. Reproduced with permission.^[^ ^314^ ^]^ Copyright 2019, American Chemical Society. c) Proposed reaction mechanism. Reproduced with permission.^[^ ^315^ ^]^ Copyright 2019, American Chemical Society.

Bortnikov and colleagues demonstrated that alternating current would be used to couple electron transfer steps at the same electrode.^[^ ^312^ ^]^ Alternating current‐assisted Ni‐catalyzed amination, esterification, and etherification of aromatic bromides result in increased selectivity and yields compared to experiments using direct current. Wang and colleagues showed that mediators, particularly those facilitating hydride and hydrogen atom transfer (HAT) reactions, significantly reduced the overpotentials necessary for the electrochemical oxidation of organic molecules (**Figure** **30**a).^[^ ^313^ ^]^ The oxidative functionalization of C—H bonds adjoining to N in piperidine derivatives as well as other concentrated heterocycles, is one of the most critical applications. Other important applications include the oxygenation and iodination of benzylic C—H bonds and the intramolecular amination of benzylic and aliphatic C—H bonds. Because of the lower operating potentials, the combinability and synthetic scope of functional groups are able to be significantly expanded. Wang et al. showed how mediators, particularly those capable of enabling hydride and HAT processes, could significantly reduce the overpotentials required for the electrochemical oxidation of organic molecules (Figure 30b).^[^ ^314^ ^]^ The hydrogen adsorption energy of the cathode material influences electron and proton transfers, which is significant for electro carboxylation efficiency. In a tiny amount of water, aromatic alcohols can be oxidized to ketones or aldehydes, followed by a transformation to hydroxy acids with yields of 61% at the cathode. Wang et al. described that the simple indivisible cell with graphene/Ni foam electrodes had exceptional substrate tolerance and produced aryl and alkyl sulfides with high chemical yields.^[^ ^315^ ^]^ Figure 30c shows the mechanism of the electrochemical cross‐coupling reaction. During the process of oxidizing the thiol at an anode, a SET reaction takes place, which results in the production of the thiol radical cation F. Pyridine proton abstraction, when combined with aryl disulfide 7, results in the production of the thiol radical G. During this time, a cathodic reduction of NiCl_2_dtbbpy A produces a Ni^0^‐X B. This is followed by the oxidative addition of aryl aide 1, which produces an Ar—Ni^2+^‐X species C. This species then captures the thiol radical G, which produces a Ni^3+^‐complex D. Finally, the reductive elimination of D results in the formation of the cross‐coupled product 3 with the Ni^1+^‐X complex E. This product is then cathodically reduced to regenerate the Ni^0^‐X B.

The electrochemical redox‐neutral cross‐coupling processes of C—Se,^[^ ^312^ ^]^ C—S,^[^ ^315^, ^316^ ^]^ C—O,^[^ ^3^, ^312^ ^]^ C—P,^[^ ^311^, ^317^ ^]^ and C—Se^[^ ^311^ ^]^ bonds have recently achieved a great deal of success using Ni‐based catalysts. Numerous electrophiles, including challenging aryl chlorides and diverse amines, can be conveniently coupled. To create Ni‐catalyzed electrochemical amination of DNA via reversible adsorption to a solid support, the groups led by Baran and Dawson took advantage of reversible adsorption to solid support and gentle reaction conditions. In their paper, a proof‐of‐concept implementation of electro‐organic synthesizing is described in biological systems that are particularly sensitive to redox chemical reagents but have limited amine substrates that are suitable for use in this context. These biological systems are also described as having restricted amine substrates that are suitable for use in this context.^[^ ^318^ ^]^ The reductive electrosynthesis of mesoporous UiO‐66‐PDC (Zr‐mMOF) was discussed in detail by Naseri and colleagues.^[^ ^319^ ^]^ It was discovered that this method of Zr‐mMOF electrosynthesis is quick, and it can be carried out without using any base or pre‐base additive for ligand activation. One of the most important and widely employed organic synthesis transformations is C—N cross‐coupling. It has established itself as a standard transformation in academia and industry, with Cu and Pd‐based catalytic systems dominating the process.

### 4.3 Convergent Paired Electrolysis

**Figure 31:**
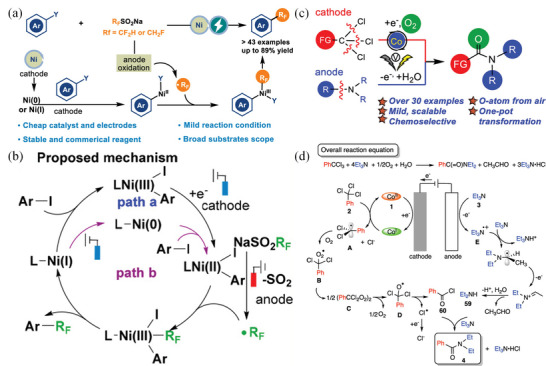
a) Iodide oxidative fluoroalkylation promoted by electrochemical activation of Ni. b) Proposed reaction mechanism. Reproduced with permission.^[^ ^325^ ^]^ Copyright 2021, American Chemical Society. c) Convergent paired electrolysis for the one‐pot synthesis of tertiary amides from organic trichlorides. d) Proposed mechanism. Reproduced with permission.^[^ ^326^ ^]^ Copyright 2021, American Chemical Society.

A convergent paired electrolysis occurs if anodically produced intermediates can react with cathodically produced intermediates. This study describes the arylation of tertiary amines and their derivatives using convergent paired electrolysis without stoichiometric oxidants or metals for synthesizing benzylic amines.^[^ ^320^ ^]^ Anodically and cathodically generated species can be cross‐coupled using this electrocatalytic approach that fully utilizes anodic and cathodic reactions. Recently, Chen and colleagues reported a paired electrochemical oxychlorination of styrenes using Mn as the catalyst for chloroacetophenone synthesis.^[^ ^321^ ^]^ As the intermediate decomposes, the benzylic alcohol is generated, and oxidized on the anode surface or with superoxide to yield the final product. This reaction is proposed to be aided by the ability of electrochemical oxygen reduction to produce carbon‐centered radicals and the cathodic reduction of oxygen to generate persistent radical anion species.^[^ ^322^ ^]^ Hu and colleagues used convergent electrochemical Ni catalysis to catalyze the direct arylation of benzylic C—H bonds by utilizing Ni's ability to participate in single‐electron radical capture mechanisms for C—C bond synthesis.^[^ ^323^ ^]^ Radicals and an electrochemical Ni catalyst form the basis of the mechanism. Upon anodic oxidation with a base, toluene derivatives become rather stable benzylic radicals, which are reductively eliminated to obtain the desired products through aryl Ni^2+^ species, which are captured by aryl Ni^3+^ species. An electrochemically‐driven Ni catalyst was used to achieve highly selective O‐arylations.^[^ ^324^ ^]^ Photochemically facilitated Ni catalysis and Pd‐facilitated Cu catalysis are state‐of‐the‐art methods for this substrate catalysis. According to the report of Zhang et al., Ni catalyzed arylation of benzylic C—H bonds via cross‐coupling of two intermediates formed at the two electrodes of the electrochemical cell.^[^ ^323^ ^]^ The method yields a wide range of diarylmethanes, which are common structural motifs in materials and medicinal chemistry. Based on preliminary mechanistic studies, the catalytic cycle appears to be composed of Ni‐catalyzed C—C coupling, oxidation of a benzylic C—H bond, and an intermediate Ni compound reduction. In order to overcome the conflict between the anodic oxidation of fluoroalkyl sulfinates and cathodic reduction of low‐valent Ni catalysts, Zou et al. used fluoroalkyl radicals with aryl halides to be cross‐coupled with metal catalysts, allowing fluorinated functionalities to be delivered directly into aromatic systems (**Figure** **31**a).^[^ ^325^ ^]^ The catalytic mechanism is described in Figure 31b. Two plausible mechanisms for the electrochemical fluoro‐alkylative coupling are proposed: either oxidative addition of Ar—I to Ni(I) forms the Ar—Ni(III)‐Rf intermediate, followed by cathodic reduction to Ni(II) (path a), or cathodic reduction of Ni(I) forms a Ni(0) species, followed by oxidative addition of Ar—I to Ni(II) (path b). The anodic oxidation of sodium fluoroalkyl sulfinate provides the fluoroalkyl radical, which is trapped by the Ar—Ni(II)‐I species to give an Ar—Ni(III)(Rf)‐I intermediate. Finally, the reductive elimination of Ni(III) gives the fluoroalkylated product and Ni(I) species.

Using a B12 complex‐mediated convergent paired electrolysis, Luo et al. synthesized a tertiary amide from organic trichlorides (R‐CCl_3_) (Figure 31c).^[^ ^326^ ^]^ Organic trichlorides are converted to amides by oxygen incorporation from air through tertiary amines in the presence of tertiary amines such as benzotrichloride and its derivatives, chloroform, dichlorodiphenyltrichloroethane (DDT), trichloro‐2,2,2‐trifluoroethane (CFC‐113a), and trichloroacetonitrile (CNCCl_3_). Mechanism research reveals that a Co complex mediates the amide formation during paired electrolysis (Figure 31d).

An improved metal‐free strategy utilizing both anodic oxidation and cathodic reduction was described by Zhang et al. for the synthesis of *α*‐benzyl amines from readily available imines and methylarenes.^[^ ^327^ ^]^ Activating the benzylic C—H bonds at the cathode to access benzylic radicals, this thiol‐mediated convergent paired electrolysis protocol reduces imines to *α*‐aminoalkyl radical species at the anode, which facilitates radical–radical cross‐coupling without the need for metals or stoichiometric oxidants. Yi, Ye, and colleagues reported using electron‐deficient aryl nitriles for TEMPO‐propelled arylation of *α*‐amino C(sp^3^)—H bonds in 2019.^[^ ^320^ ^]^ Using TEMPO as an electrocatalyst enhanced the efficiency of the reaction significantly, even though the reaction might occur without it. The convergent electrocatalytic system works with a range of tertiary arylamines; however, the ester groups on some arylamines fail to show any visible coupling reaction. Experimental findings and literary precedents point to a radical‐radical cross‐coupling mechanism. Possibly, the remarkable performance of this electrolytic system is due to its employment of a redox‐active electrocatalyst, which generates fairly stable radical reactions centered around carbon, as well as the persistence of radical character of the radical anion formed at the cathode. The authors recently reported electrochemically arylating benzylic alcohols using a batch electrochemical setup.^[^ ^328^ ^]^ The arylating reagents used again are electron‐deficient aryl nitriles, and a relatively stable coupling route based on benzylic radicals is proposed for the observed reactivities. A comparable electrochemical Ni‐catalyzed formation of C(sp^3^)—C(sp^2^) bonds was produced by Liu and colleagues by using benzoylic trifluoroborates as carbon‐centered radical precursors.^[^ ^329^ ^]^

**Figure 32:**
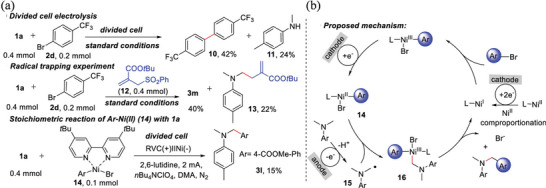
Aminomethylation of aryl bromides: a) Mechanistic studies and b) proposed mechanism. For the reaction, to the anodic and cathodic chambers were added 0.4 mmol of 1a, 0.2 mmol of 2d, 10 mol % Ni(NO_3_)_2_, 15 mol % L, 0.4 mmol of 2,6‐lutidine, 0.1 m nBu_4_NClO_4_, and 3 mL of DMA. Proposed mechanism. Reproduced with permission.^[^ ^330^ ^]^ Copyright 2021, American Chemical Society.

**Figure 33:**
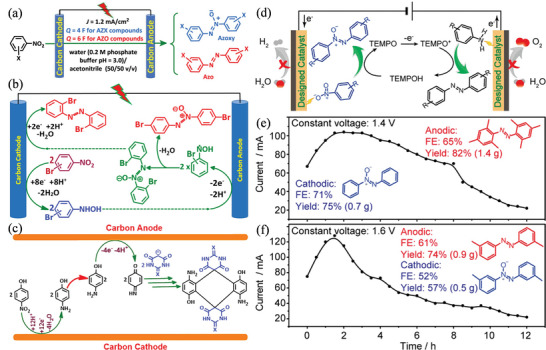
a) Convergent paired electrocatalysis of Azo and Azoxy compounds. b) Possible reaction mechanism. Reproduced with permission.^[^ ^332^ ^]^ Copyright 2021, American Chemical Society. c) An electrochemical synthesis of symmetric dispiro and spiropyrimidine derivatives based on the reduction of paranitrophenol. Reproduced with permission.^[^ ^333^ ^]^ Copyright 2021, Elsevier. d) Coupling (N—N) on Ni_3_Fe‐MOF‐OH. I–t curves for e) NB/2,4,6‐trimethylaniline and f) m‐methyl NB/m‐methyl aniline during gram scale synthesis. Reproduced with permission.^[^ ^334^ ^]^ Copyright 2021, American Chemical Society.

C‐centered radicals were produced by the anodic oxidation of trifluoroborates for Ni catalysis. The chemical system is easily scalable; however, radical stability has a problem, which limits the substrate scope to benzylic radical precursors. In addition to aryl bromides, alkenyl bromides were demonstrated to be appropriate electrophiles, while their potential applications in pharmaceuticals and natural amino acids were demonstrated in the study. Zhu et al. described the cross‐coupling amination with weak N nucleophiles such as anilines, sulfonamides, sulfoximines, carbamates, and imines, using a Ni‐catalyzed concerted paired electrolysis.^[^ ^331^ ^]^ During the arylation of benzophenone imine, a base switch can be applied to alter the selectivity of the product toward the formation of amine or imine. An electrochemical method for the aminomethylation of aryl bromides has been developed by Ma and colleagues (**Figure** **32**a).^[^ ^330^ ^]^ During convergent paired electrolysis, cathode and anode processes are fully utilized without terminal oxidants, sacrificial anodes, metal reductants, and pre‐functionalized radical precursors. In addition to some sensitive substituents and aromatic heterocycles, the method is highly tolerable to functional groups. The catalytic mechanism is described in Figure 32b. Initially, Ni(II)L undergoes a two‐electron reduction process at the cathode to afford Ni(0)L, which undergoes a rapid Ni(0)/Ni(II) comproportionation process to form Ni(I)L. The following oxidative addition of Ni(I)L to aryl bromide forms an Ar‐Ni(III)L‐Br intermediate, which is reduced to Ar‐Ni(II)L‐Br (14) at the cathode. Meanwhile, *α*‐amino carbon radical 15 is produced after oxidation at the anode and deprotonation. In addition, the formation of 15 might involve the cathodic reduction of iminium, the overoxidized product of 15. In bulk solution, 14 rapidly captures 15, (24), generating the high‐valent Ni(III) adduct 16. Subsequent reductive elimination of 16 produces the coupling product and Ni(I) complex. Another possible pathway involved in the Ni(0)/Ni(II)/Ni(III) catalysis cannot be ruled out at present. Jensen and Buchwald have demonstrated a microfluidic electrochemistry system to produce redox‐neutral radical reactions in 2020 without the flaws of batch electrolysis reactors.^[^ ^316^ ^]^ Species formed at the cathode and anode may be selectively coupled to produce the required products. The anodic oxidation of carboxylic acids, *α*‐amino C(sp^3^)—H bonds, and potassium organoborates generates radicals with C atoms at their center. In order to form C—C bonds, the radical anion intermediates can be coupled with relatively stable radical anion intermediates generated at the cathode. Sadatnabi et al. have developed a method based on paired electrochemical reactions for synthesizing nitroarenes into azoxy and azo compounds (**Figure** **33**a).^[^ ^332^ ^]^ An undivided cell is successfully used to synthesize azoxy and azo derivatives via constant current electrolysis with carbon rod electrodes (Figure 33b).

Using a new type of green electrochemical method, Khoram et al. synthesized pyrimidine derivatives containing spiro and dispiro rings (Figure 33c).^[^ ^333^ ^]^ *P*–nitrophenol is electrochemically converted into reactive intermediates, and the reactions with barbituric acids are essential for the production of target products. Qiao et al. demonstrated the coupled reduction of nitroaromatics to azoxy‐aromatics and the oxidative reduction of aromatic amines to azo‐aromatics by using a surface hydroxylated Ni_3_Fe‐MOF‐OH bifunctional electrocatalyst (Figure 33d) with excellent efficiency and selectivity.^[^ ^334^ ^]^ Despite its high stability, Ni_3_Fe‐MOF‐OH can be used to synthesize azo‐ and azoxy‐aromatic compounds (Figure 33e,f) with good yields and FE, making it an attractive synthetic tool.

## 5 Conclusions and Prospects

The continued price declines of renewable electricity and natural gas open many possibilities for electrochemical oxidation and reduction of low‐priced organic compounds into high‐value products. Comparatively to conventional organic synthesis technologies, electrochemical conversion can be run intermittently and modularly at a wide range of temperatures and pressures to enable highly distributed production of products. The research efforts in this key area are still lacking, and the technology is still in its infancy and far from widespread commercialization. Our review summarizes all recent developments in electrochemical reduction and oxidation processes with heterogeneous catalysts and a brief analysis of the technological and economic issues involved. HER/OER is replaced by electro‐organic reduction/oxidation, which is thermodynamically more advantageous and yields a higher‐valued product. However, stability, efficiency, product selectivity, mediator separation/isolation, and a theoretical understanding of cathodic/anodic processes are the hurdles that must be overcome. If it is to be commercialized, future research on electro‐organic synthesis should be focused on the following important aspects.

Commercialization of heterogeneous electrocatalysis requires cost‐effective approaches for synthesizing stable, earth‐abundant electrocatalysts. Ni, Co, and Fe are among the non‐noble earth elements that can be used as bifunctional electrocatalysts. However, the activity and selectivity still need to improve. The slow dissolution of the catalysts in these harsh conditions is detrimental to catalyst stability. With highly concentrated alkali solutions, cathodic/anodic reactions minimize ionic losses and facilitate oxidation and reduction. Therefore, it is necessary to explore earth‐abundant electrocatalysts for their high catalytic performance and stability across a wide pH range.

Most recent reports on anodic oxidation reactions focus on biomass degradation, alcohols, ammonia, and urea oxidation. Although alcohol oxidation in combination with HER or eCO_2_RR has been extensively studied, it is still in its early stages of development and a long way from being competitive with petroleum‐derived hydrogen. As‐synthesized products require complicated techniques and expensive procedures to be separated and purified from electrolytes. To fully commercialize complex alcohol oxidation, better reaction conditions are needed to complete product selectivity and purification. A number of oxygenated platform molecules can also be synthesized from hydrocarbon feedstocks by electrochemically activating C—H.

Operand techniques must be employed to understand electrochemical oxidation and catalytic surface changes at the cathode and anode during electrochemical reactions. There are a number of electro‐organic conversions emerging at a rapid pace. In most cases, CV is necessary to verify the postulated mechanism.^[^ ^335^ ^]^ However, CV only measures electron transfers at the interface between solids and liquids. This results in a lack of structural sensitivity because it is not accessible to chemical processes. Metal atoms inside the electrocatalyst must be detected using spectroelectrochemistry to determine the kind and shape of the active site as well as the adsorption and desorption of intermediates. In situ techniques such as Fourier transform infrared spectroscopy, Raman, X‐ray powder diffraction, and X‐ray photoelectron spectroscopy can be applied to detect electro‐oxidation pathways and analyze catalyst surface change.

A hybrid electrolyzer in a microflow cell design offers a more significant amount of substrate to the catalyst layer, allowing it to be activated without reaching the limit of mass transport, as opposed to traditional H‐cell reactors. As a result, the use of GDLs with catalyst coatings is on the rise, which enables high substrate concentrations to be maintained at the catalyst layer despite high reaction rates when a peristaltic pump is used. In addition to stabilizing the triple‐phase boundary between solid catalyst, liquid electrolyte, and CO_2_ gas with eCO_2_RR, GDLs improved direct catalysis to reduce overall cell potential. A paired electrolysis process can increase productivity using micro‐flow cells and a better ratio between electrolyte flow and electrode area.

Having examined several aspects of electro‐organic synthesis, we can conclude that it is a promising technique with many discoveries, but one with plenty of potential hidden treasures that will lead to its further development. The ability to make value‐added chemicals using fluctuating and abundant electricity will dramatically impact chemical operations in the future. In order to achieve sustainable chemistry, organic chemical synthesis must master these challenges.

## Conflict of Interest

The authors declare no conflict of interest.
